# Evaluation of a highly stable biogenic palladium nanocatalyst for industrially relevant and sustainable nitro reduction

**DOI:** 10.1039/d6na00568c

**Published:** 2026-09-28

**Authors:** Hemal B. Rathod, Yashasvi N. Desai, Paresh N. Patel

**Affiliations:** a Laboratory of Bio-Organic Chemistry, Tarsadia Institute of Chemical Science (TICS), Uka Tarsadia University Bardoli – 394 350 Gujarat India pareshn111@yahoo.com

## Abstract

The catalytic reduction of nitroarenes to aromatic amines is an important transformation for environmental remediation and industrial production of valuable chemicals. In this study, we report the use of a biogenic palladium nanoparticle (AtPdNP) synthesized by the *Aspergillus trinidadensis* VM ST01′ OL587588 fungus for the highly efficient and selective reduction of various nitro compounds. The reduction reactions are successfully carried out in a green aqueous medium at room temperature under mild conditions with sodium borohydride (NaBH_4_) as the hydride source. The AtPdNP catalyst shows good functional group tolerance over a broad range of aromatic ring systems, including monocyclic, bicyclic, polycyclic and heterocyclic scaffolds. The catalyst exhibits excellent conversions (90–100%) and high isolated yields (80–95%) within short reaction times of 30 to 60 min. Additionally, the AtPdNP shows remarkable chemoselectivity, which enabled the efficient reduction of halogen-substituted nitroarenes without any unwanted side reaction of dehalogenation. Importantly, it shows excellent chemoselectivity between ketone/aldehyde and nitro groups by reducing only the nitro functionality using hydrogen gas. Besides catalytic efficiency, the heterogeneous AtPdNP demonstrates outstanding practical stability with high catalytic activity (>90% conversion and >80% isolated yields) throughout 10 successive reaction cycles. The catalyst also retains its intrinsic activity over a 12 month storage period under ambient conditions. Extreme thermo-mechanical stress studies show no leaching of palladium into the aqueous phase, demonstrating the strong anchoring of Pd nanoparticles within the fungal biomass matrix. Overall, the AtPdNP catalyst is a robust, sustainable and highly reliable heterogeneous platform for selective reduction of nitroaromatics.

## Abstract 2

The catalytic reduction of nitroarenes to aromatic amines is an important transformation for environmental remediation and industrial production of valuable chemicals.

## Graphical abstract

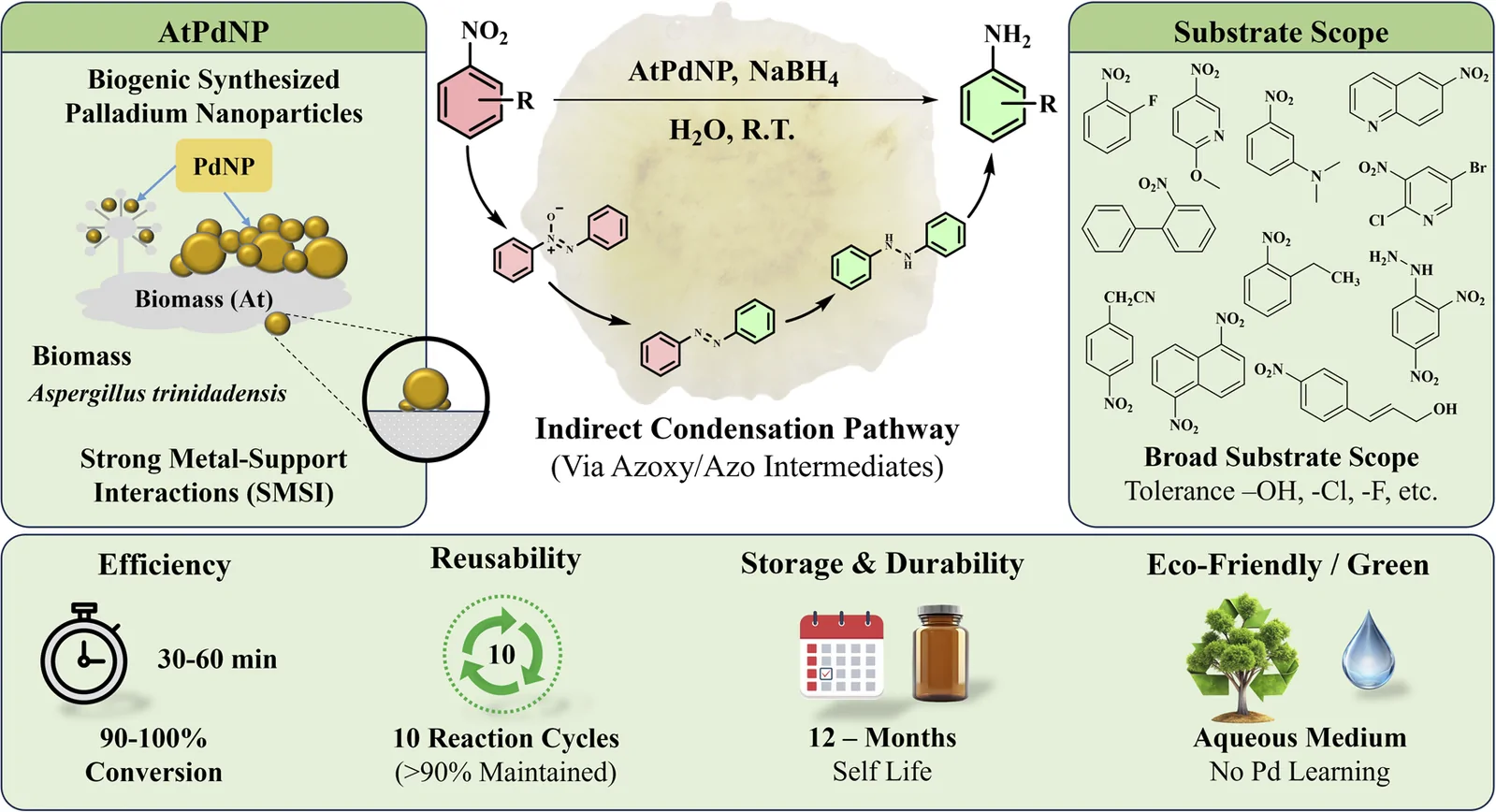

## 1 Introduction

Nitroaromatic chemicals are extensively utilized in industrial applications.^1^ However, they are recognized as persistent carcinogenic pollutants and toxic bio-refractories that pose significant risks to aquatic ecosystems and human health.^1,2^ These compounds have high stability and it is extremely difficult to degrade them completely.^2^ Thus, catalytic reduction of nitroarenes to their corresponding amino derivatives has been a strategic and very effective approach.^3^ The transformation not only diminishes the environmental hazards associated with nitro compounds but also produces aromatic amines, which are important intermediates for the manufacture of medicines, agrochemicals, dyes, polymers and fine chemicals.^2,4,5^ The traditional routes for the reduction of nitro compounds require severe conditions such as high temperature and pressure or the use of hazardous reagents, which generate secondary waste. To avoid these disadvantages, researchers have turned to catalytic transfer hydrogenation, using reducing agents such as sodium borohydride (NaBH_4_) in aqueous media. In this respect, heterogeneous catalysts are highly desirable over homogeneous catalysts. Homogeneous systems suffer from recovery problems, limited reusability and the risk of heavy-metal contamination of the end products. Heterogeneous catalysts, on the other hand, are easy to separate, highly recyclable and stable, and therefore highly applicable for sustainable and industrial-scale production.^4,6–15^

The reduction of nitroaromatics has been studied using several heterogeneous catalytic systems, in which noble metal nanoparticles have been shown to be highly efficient.^16,17^ Palladium (Pd), in particular, stands out as a premier catalytic metal due to its remarkable hydrogen adsorption and activation capabilities.^4,7,11,12,18^ Palladium based nanocatalysts have high atomic efficiency, unique electronic structures and a great capability for promoting selective reduction pathways. Nanoscale palladium has a high surface area to volume ratio, which provides many active sites, which in turn causes a significant acceleration of the reaction kinetics, compared to bulk metals.^19,20^ The catalytic reduction of nitro compounds over palladium nanoparticles generally proceeds *via* a Langmuir–Hinshelwood or an electron-relay mechanism.^21^ In the presence of a reducing agent like NaBH_4_, the Pd nanoparticles act as a good electron transport system. Co-adsorption of the hydride source and the nitroarene molecules on the active palladium surface is observed. The palladium nanoparticles then ensure a rapid transfer of electrons and hydride species from the hydride source to the electrophilic nitro group *via* the nitroso and hydroxylamine intermediates or 1,2-diphenyldiazene 1-oxide and azobenzene intermediates, leading to the final amine product.^9,22^ The Pd active sites greatly lower the activation energy barrier and ensure rapid and complete conversion.

Though the catalytic activity of palladium nanoparticles is better, the practical application is often hindered by the high cost of the materials and their strong tendency to agglomerate and leach during the catalytic process. The aggregation results in a dramatic decrease of the available surface area and consequently compromises the catalytic efficiency. To overcome these problems, stabilization of Pd nanoparticles on suitable heterogeneous supports is an important approach.^10,15^ The ideal support matrix should immobilize the nanoparticles to prevent agglomeration and leaching, as well as enhance atomic-level dispersion to maximize the exposure of active sites.^10,15^ Moreover, the interaction between the palladium nanoparticles and the heterogeneous support can alter the electronic structure of the metal to adjust the adsorption of the reactant substrate to improve the overall catalytic turnover.

Such catalysts play a central role in modern chemical industries by enabling chemical transformations with improved reaction rates, selectivity, energy efficiency and reduced waste generation. However, high catalytic activity alone is insufficient to establish the industrial viability of a catalyst.^16,23^ Comprehensive evaluation should include catalytic efficiency and productivity, substrate scope, reaction kinetics, catalyst loading, tolerance toward different functional groups, chemoselectivity and performance under practically relevant reaction conditions.^16,17^ In addition, catalyst stability, reusability over multiple cycles, long-term storage stability, resistance to deactivation, metal leaching and structural integrity after repeated use are essential parameters for assessing operational durability.^19,20^ Investigation of different hydrogen sources and reaction safety can further establish practical advantages.^23,24^ Scale-up experiments, catalyst reuse, product yield-purity, material and energy requirements and preliminary economic and environmental assessments are also important for determining process feasibility.^25^ Collectively, these studies provide a comprehensive basis for demonstrating whether a catalyst can be translated from laboratory-scale research to a robust, sustainable, economically viable, and industrially applicable catalytic process.

Guided by these principles, the present work highlights the use of our previously prepared and well characterised palladium-based nanoparticles^26^ as a high-performance heterogeneous catalyst for the selective reduction of nitro compounds. This research aims to highlight the efficiency of the industrially viable catalyst under mild conditions by investigating the catalytic activity with various hydride sources, chemoselectivity, storage stability, kinetic behaviour and reusability of the supported palladium system in the reduction of nitroarenes. The catalytic performance is discussed in the following sections, and its potential as a sustainable, robust and highly efficient platform for environmental remediation of nitroaromatics and production of valuable aromatic amines is evaluated.

## 2 Experimental

### 2.1 Chemicals and characterization techniques

Palladium(ii) chloride (Reagent Plus®, 99%) was purchased from Sigma-Aldrich. All solvents of LR and HPLC grade were obtained from Rankem through local suppliers. Nitro-substrates used in this study were procured from local vendors and were manufactured by Sigma-Aldrich, Rankem, SpectroChem, and Tokyo Chemical Industry (TCI). The conversion of nitro substrates to their corresponding amines was quantitatively determined using a Shimadzu Prominence-i LC-2030 High-Performance Liquid Chromatography (HPLC) system. The formation of the corresponding amino derivatives was further confirmed by Agilent Technologies single Quadrupole LC/MS 6120 liquid chromatography-mass spectrometry (LC-MS) analysis through the observation of the expected molecular ion peaks corresponding to the reduced amine products. The combined HPLC and LC-MS analyses verified both the conversion and identity of the synthesized amines. The ^1^H NMR spectra were recorded in CDCl_3_ or DMSO-d6 at room temperature using a Bruker AVANCE III 500 MHz (AV 500)/Bruker AVANCE III 400 MHz (AV 400) multinuclear solution NMR spectrometer. TMS was used as an internal reference. The spectra were characterized by integration, multiplicity (s = singlet, d = doublet, t = triplet, q = quartet, quin = quintet, m = multiplet, br = broad, and app = apparent), coupling constants (*J*; Hz), and assignment. ^13^C and DEPT-135 NMR spectra were recorded on a Bruker AVANCE III 125 MHz (AV 125)/Bruker AVANCE III 100 MHz (AV 100) with complete proton decoupling. Chemical shifts were reported in ppm from the residual solvent as an internal standard.

### 2.2 Preparation and inclusive characterization of AtPdNP^26^

In our previous study, we established a sustainable one-pot biosynthetic approach for the preparation of palladium nanoparticles (AtPdNP) using *Aspergillus trinidadensis* VM ST010 OL587588 as a biological reducing and stabilizing system. The synthesis process was systematically optimized with respect to fungal culture age, biomass concentration, palladium precursor concentration, and incubation conditions, and the resulting AtPdNP catalyst was comprehensively characterized using UV-Vis spectroscopy, FT-IR, XPS, powder X-ray diffraction, SEM, TEM, EDX with elemental mapping, TGA/DSC/DTA, and zeta-potential analysis. These investigations confirmed the successful formation of Pd nanoparticles within the fungal biomass matrix, with TEM revealing nanoparticles predominantly in the 20–40 nm range and an average particle diameter of 30.33 ± 3.56 nm. The FT-IR and XPS analyses further supported the involvement of biomass-derived functional groups in the stabilization of the Pd nanoparticles and the formation of Pd(0), while EDX confirmed the incorporation of palladium within the biogenic matrix. Building upon this established and comprehensively characterized catalyst, the present study does not involve a new synthesis or redesign of AtPdNP. Instead, the previously optimized catalyst was employed directly to undertake an expanded investigation of its catalytic performance, including substrate scope, reduction efficiency, chemoselectivity, robustness, reusability, storage stability, metal leaching and industrial applicability. Thus, the present work presents a systematic catalytic application and viability study of the established biogenic AtPdNP catalyst.

Here, *Aspergillus trinidadensis* VM ST010 OL587588 acts as a biological reducing and capping system, in which biomolecular functional groups associated with the fungal biomass facilitate the reduction of Pd^2+^ to Pd^0^, nucleation of Pd nanoparticles, and subsequent surface stabilization of the formed nanoparticles within the biomass matrix.^9,22,26,27^

### 2.3 General procedure for nitro reduction using the AtPdNP catalyst

A dried sample of the AtPdNP catalyst (5 mg) was dispersed in 5 mL of double-distilled water and transferred to a 25 mL round-bottom flask (RBF) containing 1 mmol of the nitro derivative. The reaction flask was placed in an ice bath and maintained at 0–5 °C. Subsequently, NaBH_4_ (2 mM) was added as a hydride source, and the flask was tightly sealed to prevent the escape of hydrogen gas generated during the reaction. After 5 min, the ice bath was removed, and the reaction mixture was stirred continuously at room temperature. The progress of the reduction was monitored by thin-layer chromatography (TLC) using ninhydrin staining for visualization. Upon completion of the reaction, 5 mL of ethyl acetate was added, and the mixture was thoroughly stirred to facilitate extraction of the product. The reaction mixture was then allowed to stand undisturbed to achieve phase separation. The organic layer was collected and dried over anhydrous sodium sulphate. Removal of the solvent under reduced pressure afforded the corresponding amino derivative (Scheme 1). The recovered catalyst was separated, washed thoroughly with distilled water, and reused in subsequent catalytic cycles to evaluate its reusability.^9,22,26^

**Scheme 1:**
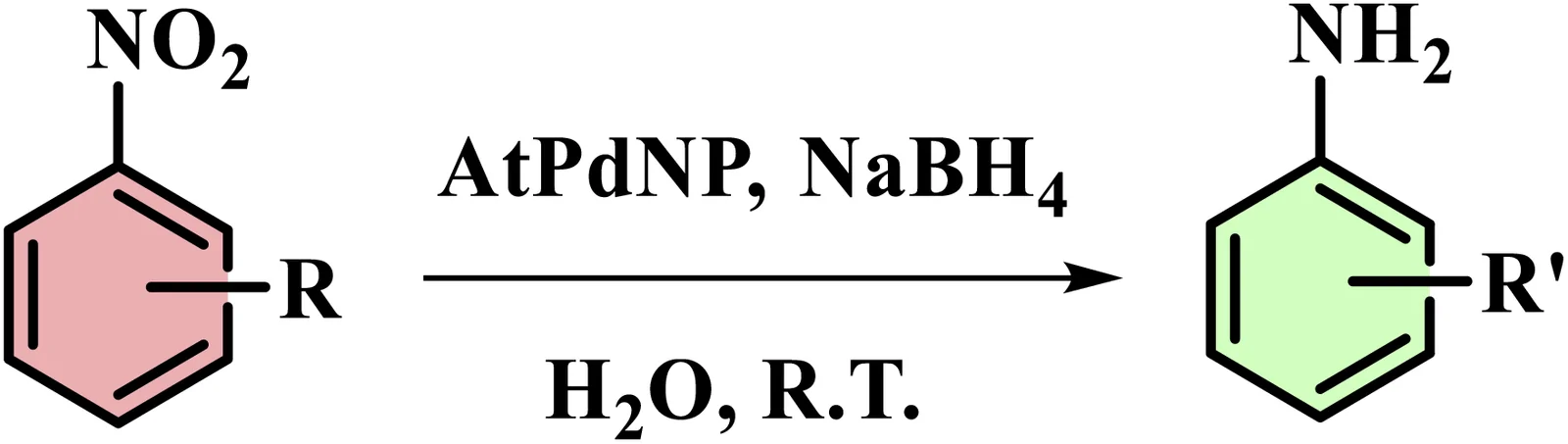
General scheme for nitro reduction by AtPdNP.

## 3 Results and discussion

### 3.1 Optimization of nitro group reduction using a model reaction using AtPdNP

Nitrobenzene was selected as the model substrate to evaluate the catalytic performance of the AtPdNP catalyst. The reaction was performed in water as a green solvent at room temperature under mild conditions. The reduction of nitrobenzene was carried out following the general reduction procedure described above, using 1 mmol of nitrobenzene as the substrate. All reaction parameters, including catalyst loading, sodium borohydride concentration, reaction temperature, and reaction time, were optimized using this model reaction. The optimized conditions obtained from the nitrobenzene reduction were subsequently applied to the reduction of other nitro derivatives to assess the substrate scope and catalytic efficiency of the AtPdNP catalyst (Scheme 2).

**Scheme 2:**
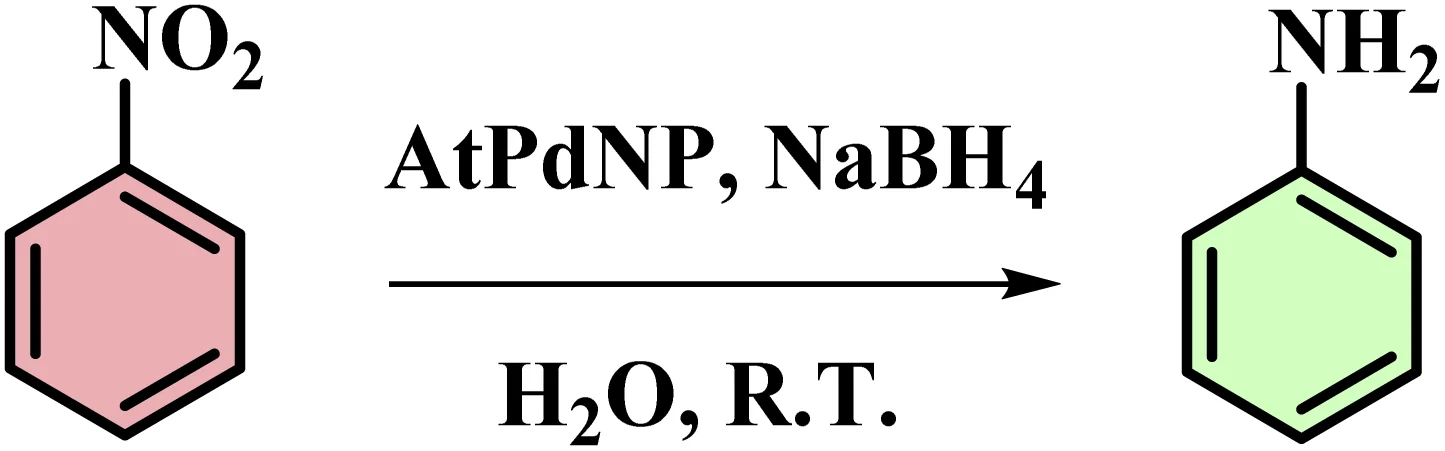
Model substrate reaction for nitro reduction.

In order to gain a full understanding of the reaction mechanism and the functions of the various components, some control experiments were performed. These included reactions with/without the catalyst, the hydride source, both the catalyst and hydride source, and the starting material (nitrobenzene). A blank reaction was performed without any of the three components. These control conditions confirmed the importance of each component in the reduction process in general.^26^

The AtPdNP catalyst and a fixed reaction time of 4 h were employed for all entries presented in Table 1. The optimization focused on determining the appropriate molar ratio of sodium borohydride (NaBH_4_), palladium catalyst, and nitrobenzene. Initially, the palladium loading was fixed at 5 µmol, while the concentration of NaBH_4_ was systematically varied. At lower NaBH_4_ concentrations, the reduction of nitrobenzene did not proceed to completion, and the reaction predominantly yielded azobenzene as an intermediate product. As the NaBH_4_ concentration was increased, the formation of aniline was observed, indicating further reduction of the intermediate species. Complete conversion of nitrobenzene to aniline was achieved at 2 mmol of NaBH_4_, which was therefore selected as the optimum hydride concentration for subsequent studies. Following optimization of the NaBH_4_ loading, its concentration was fixed at 2 mmol, and the palladium loading was varied from 5 µmol to 2.5 µmol. Comparable conversion efficiencies were obtained under both catalyst loadings, demonstrating that the lower palladium concentration was sufficient to achieve effective catalytic reduction. These results indicate the high catalytic efficiency of the biogenic AtPdNP catalyst, enabling a significant reduction in catalyst usage without compromising product yield (Table 1).

**Table 1:** Nitrobenzene reduction using the prepared AtPdNP (palladium & NaBH_4_ eq. optimization)

Sr. No.	Palladium (µmol) in AtPdNP	NaBH_4_ (mmol)	Reaction time	Conversion (%)
1	5	0.5	4 h	5%
2	5	1	4 h	30%
3	5	1.5	4 h	80%
4	5	2	4 h	100%
5	3.9	2	4 h	100%
**6**	**2.5**	**2**	**4 h**	**100%**

To determine the optimum reaction time for the reduction of nitrobenzene catalyzed by AtPdNP, the reaction was monitored at different time intervals, and the results are summarized in Table 2. Among Entries 1–5, Entry 3 was identified as having the most suitable reaction time for the catalytic system. Although Entries 1 and 2 exhibited complete conversion (100%), they required longer reaction times and therefore offered no practical advantage. In contrast, Entries 4 and 5 resulted in lower conversions of 85% and 50%, respectively, indicating insufficient reaction time for complete reduction.

**Table 2:** Nitrobenzene reduction using the prepared AtPdNP (reaction time optimization)

Sr. No.	AtPdNP type	Reaction time	Conversion (%)
1	AtPdNP	2 h	100%
2	AtPdNP	1 h	100%
**3**	**AtPdNP**	**30 min**	**100%**
4	AtPdNP	15 min	85%
5	AtPdNP	10 min	50%

### 3.2 Investigation of substrate versatility in the AtPdNP catalysed nitro reduction process

To comprehensively evaluate the catalytic efficiency and practical applicability of the biogenic AtPdNP catalyst, the substrate scope was investigated using a diverse range of nitroarene derivatives. The catalytic reductions were performed systematically under environmentally benign and mild reaction conditions, employing sodium borohydride (NaBH_4_) as the hydrogen source in water as the reaction medium at room temperature. A series of nitroarenes bearing electron-donating and electron-withdrawing substituents at different positions on the aromatic ring were subjected to the optimized reaction conditions (Tables 3 and 4). The reduction reactions were conducted using 1 mmol of the respective nitroarene substrate, and the reaction progress was monitored by High-Performance Liquid Chromatography (HPLC). Substrate conversion was determined from the corresponding chromatographic peak areas, while the formation of the desired amine products was further confirmed by LC-MS analysis. Upon completion of the reactions, the products were isolated through standard extraction procedures and, when necessary, purified by column chromatography. The isolated yields of the corresponding aromatic amines were subsequently determined. The results demonstrate the broad substrate tolerance of the AtPdNP catalyst and highlight its effectiveness for the selective reduction of nitroarenes under green and sustainable reaction conditions.

**Table 3:** Substrate versatility on the monocyclic aromatic ring for nitro reduction^a^

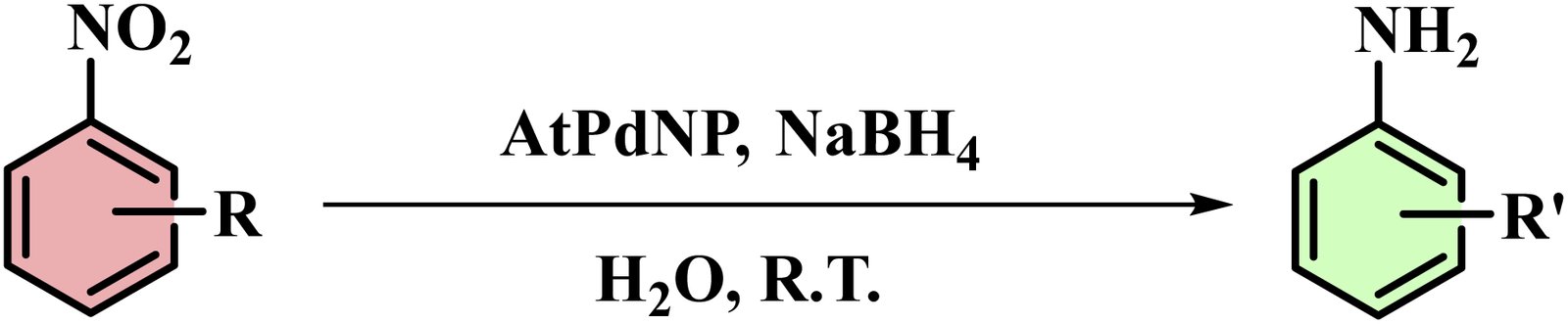						
Sr. No.	Substrate (R)	Product (R′)	NaBH_4_ (eq.)	Reaction time (min)	Conversion^b^ (%)	Yield^c^ (%)
1	4-OH	4-OH	2	30	100	95.20 ± 0.55
2	2-OH	2-OH	2	30	100	95.54 ± 0.49
3	3-OH	3-OH	2	60	98	93.09 ± 1.03
4	4-NH_2_	4-NH_2_	2	30	100	95.02 ± 0.99
5	2-NH_2_	2-NH_2_	2	30	100	95.08 ± 0.74
6	3-NH_2_	3-NH_2_	2.5	60	94	90.71 ± 0.68
7	4-Cl	4-Cl	2	50	98	92.33 ± 0.75
8	3-Cl	3-Cl	2	30	100	95.24 ± 1.06
9	2-Cl	2-Cl	2	50	98	92.02 ± 0.58
10	2-F	2-F	2	60	99	95.00 ± 1.07
11	4-F	4-F	2	60	100	95.60 ± 0.58
12	3-NO_2_	3-NH_2_	2	30	100	95.44 ± 0.81
13	2-NHNH_2_, 5-NO_2_	2-NHNH_2_, 5-NH_2_	2	30	100	95.02 ± 0.79
14	3-N(CH_3_)_2_	3-N(CH_3_)_2_	2.5	150	90	85.35 ± 1.01
15	4-CH_2_CN	4-CH_2_CN	2	30	100	95.28 ± 0.06
16	2-CH_2_CH_3_	2-CH_2_CH_3_	2	60	100	95.23 ± 0.33
17	4-CH_3_	4-CH_3_	2	60	100	95.41 ± 1.04
18	4-COOH	4-COOH	2	90	50	—
19	4-COCH_3_	4-CHOHCH_3_	2	30	100	95.84 ± 1.10
20	4-CHO	4-CH_2_OH	2	30	100	95.52 ± 0.31
21	2-CHO	2-CH_2_OH	2	30	100	95.30 ± 0.64

a 1 mmol nitroarenes.

b Conversion based on HPLC data.

c Yield after workup or column chromatography.

**Table 4:** Substrate versatility on bicyclic, polycyclic & heterocyclic aromatic rings for nitro reduction^a^

Sr. no.	Substrate (R)	Product (R′)	NaBH_4_ (eq.)	Reaction time (min)	Conversion^b^ (%)	Yield^c^ (%)
1	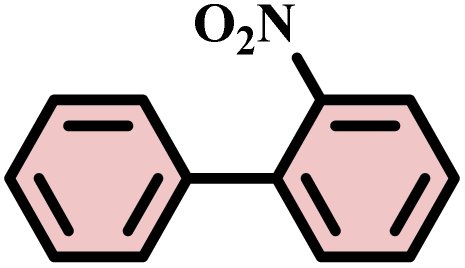	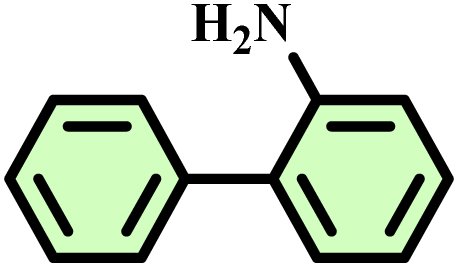	2	60	91	80.73 ± 0.45
2	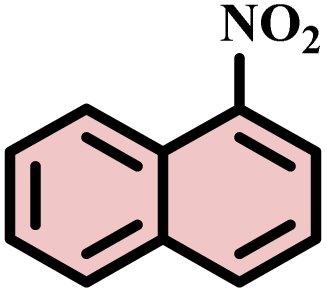	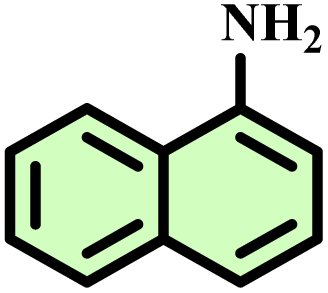	2	30	100	95.15 ± 0.80
3	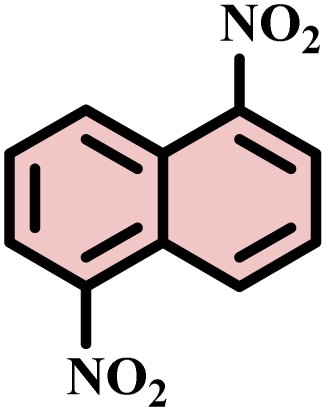	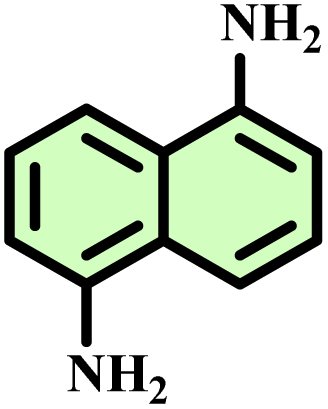	2	60	90	80.22 ± 0.24
4	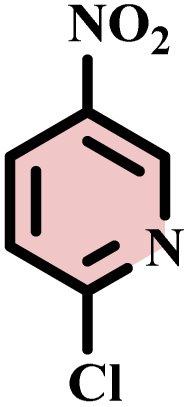	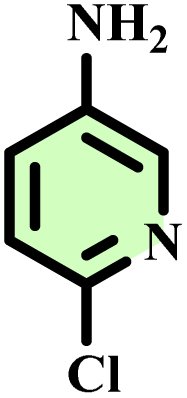	2	30	97	90.34 ± 0.60
5	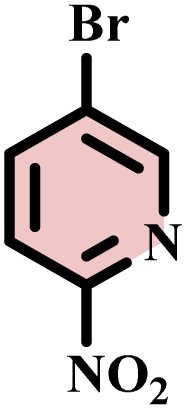	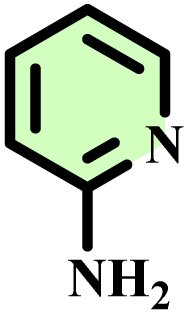	2	30	100	95.26 ± 0.45
6	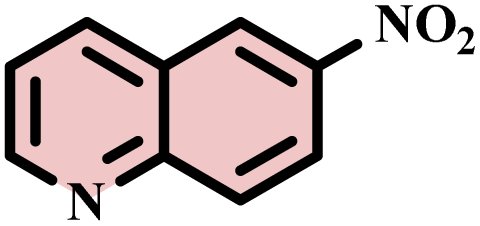	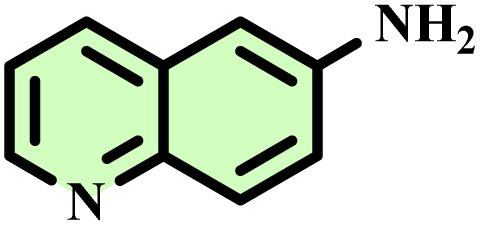	2	30	100	95.43 ± 0.80
7	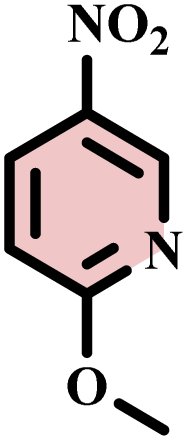	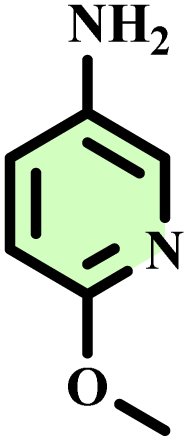	2	30	100	95.16 ± 0.80
8	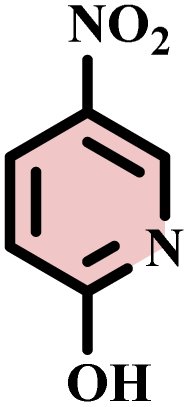	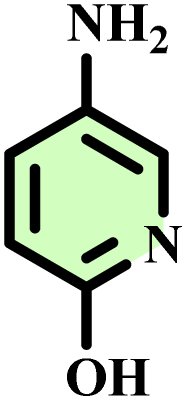	2	30	100	95.32 ± 0.47
9	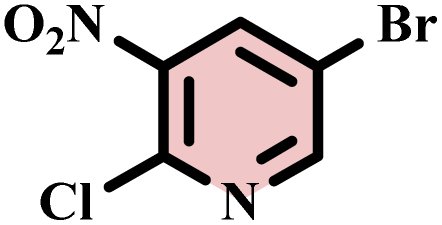	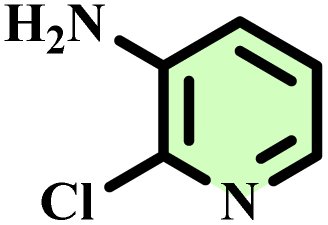	2	60	94	85.16 ± 0.64
10	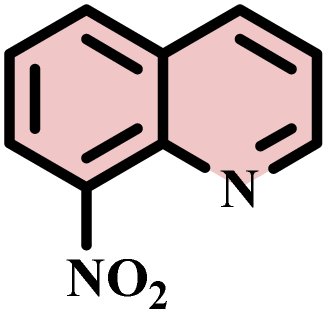	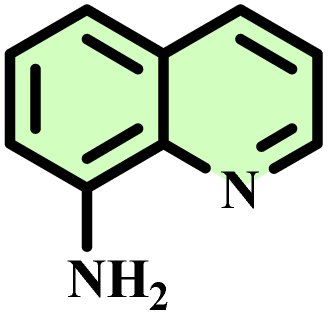	2	30	100	95.08 ± 0.63
11	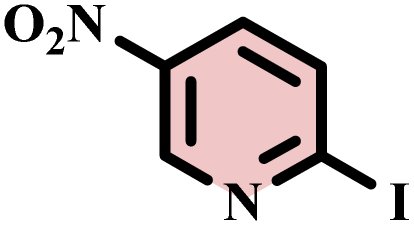	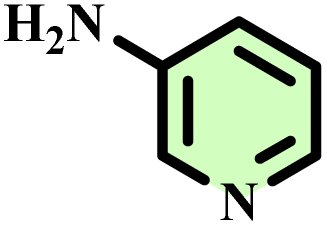	2	30	100	95.17 ± 0.16
12	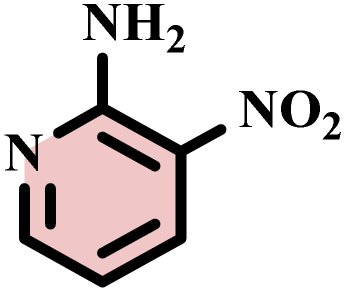	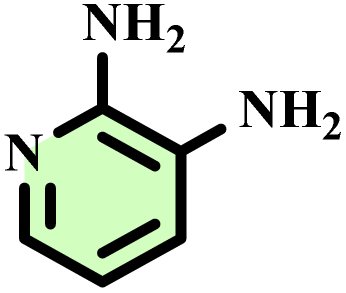	2	30	100	95.02 ± 0.20
13	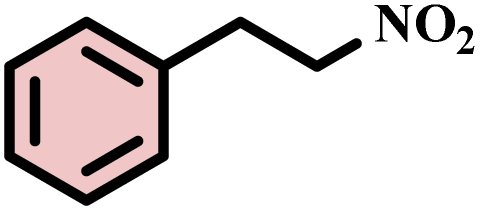	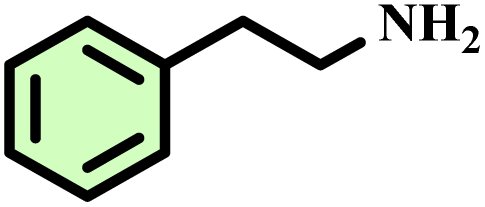	2	30	92	85.12 ± 0.41
14	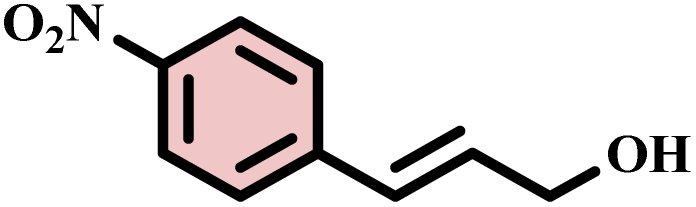	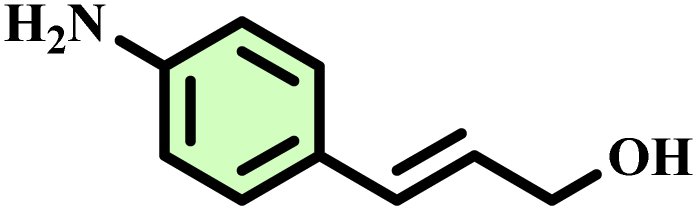	2	30	95	85.23 ± 0.19

a 1 mmol nitroarenes.

b Conversion based on HPLC data.

c Yield after workup or column chromatography.

#### 3.2.1 Monocyclic aromatic rings

The catalytic performance of the AtPdNP catalyst was evaluated using a broad range of substituted nitroarenes, and the results are summarized in Table 3. Overall, the catalyst exhibited excellent activity, functional-group tolerance, and chemoselectivity under the optimized reaction conditions. Most substrates underwent efficient reduction within 30–60 min using only 2–2.5 equivalents of NaBH_4_, affording the corresponding amines in high to excellent conversions and isolated yields. Nitroarenes bearing electron-donating substituents, such as hydroxyl (–OH) and amino (–NH_2_) groups at the *ortho*, *meta*, and *para* positions, were readily reduced to the corresponding aminophenols and phenylenediamines, providing near-quantitative conversions (94–100%) and excellent isolated yields (>90–95%). These results demonstrate that the presence of strongly electron-donating groups does not adversely affect the catalytic activity of AtPdNP. A notable feature of the catalytic system is its excellent chemoselectivity toward halogen-substituted nitroarenes. Substrates containing fluorine and chlorine substituents at different positions of the aromatic ring (2-F, 4-F, 2-Cl, 3-Cl, and 4-Cl) were efficiently converted to the corresponding haloanilines with conversions of 98–100% and isolated yields exceeding 92–95%. Importantly, no evidence of dehalogenation was observed, highlighting the ability of the AtPdNP catalyst to selectively reduce the nitro group while preserving carbon–halogen bonds. The AtPdNP/NaBH_4_ system also displayed tandem reduction capability toward substrates containing additional reducible functional groups. Nitroarenes bearing alkyl substituents, including methyl (4-CH_3_), ethyl (2-CH_2_CH_3_), cyanomethyl (4-CH_2_CN), and dimethylamino [3-N(CH_3_)_2_] groups, were smoothly converted into the corresponding aniline derivatives with high conversions and isolated yields (>85–95%). Furthermore, carbonyl-containing substrates underwent simultaneous reduction of both nitro and carbonyl functionalities. For example, 4-nitroacetophenone (4-COCH_3_) was quantitatively converted to 4-(1-hydroxyethyl)aniline, while 2- and 4-nitrobenzaldehydes (2-CHO and 4-CHO) afforded the corresponding aminobenzyl alcohols within 30 min, demonstrating the multifunctional reducing capability of the catalytic system. Substrates containing multiple reducible nitrogen functionalities were also effectively transformed. Dinitro- and hydrazine-substituted aromatic compounds were completely reduced to the corresponding polyamino derivatives, furnishing high conversions and isolated yields (>95%). These results further confirm the robustness and versatility of the AtPdNP catalyst toward structurally diverse substrates. In contrast, 4-nitrobenzoic acid exhibited significantly lower reactivity under the optimized conditions. Only 50% conversion was achieved after a reaction time of 90 min. The reduced efficiency is likely attributable to the presence of the strongly acidic carboxylic acid group, which may partially neutralize the basic reaction medium, promote competing side interactions, or inhibit catalytic activity through adsorption on the palladium surface. Nevertheless, the catalyst maintained good performance across the majority of substrates examined, underscoring its potential as an efficient and sustainable catalytic system for the reduction of nitroarenes in aqueous media.

#### 3.2.2 Polycyclic, bicyclic, and heterocyclic aromatic rings

To further assess the robustness and substrate versatility of the AtPdNP catalyst, its catalytic activity was examined using more sterically demanding and structurally complex nitroaromatic substrates, including bicyclic, polycyclic, and heterocyclic aromatic nitro compounds (Table 4). Remarkably, the increased steric bulk and extended aromatic frameworks of these substrates had little to no adverse effect on the catalytic performance. Under the optimized reaction conditions, employing only 2 equivalents of NaBH_4_, the complex nitroaromatic substrates underwent efficient reduction to their corresponding amines, affording complete or near-complete conversions (90–100%) and excellent isolated yields (>80–95%). Furthermore, despite their larger molecular size and increased structural complexity, the reactions were completed within relatively short time periods of 30–60 min under ambient conditions.

The high catalytic efficiency observed for these substrates suggests that the active palladium sites on the AtPdNP surface remain readily accessible, facilitating rapid mass transfer and effective substrate–catalyst interactions. Consequently, steric hindrance associated with bulky bicyclic and polycyclic aromatic systems does not significantly impede the reduction process. These findings highlight the excellent surface accessibility and catalytic adaptability of the biogenic palladium nanocatalyst. Overall, the heterogeneous AtPdNP catalyst demonstrates exceptional robustness, broad substrate scope, and high catalytic efficiency for the reduction of nitro compounds. Its ability to operate effectively at room temperature in aqueous media, combined with its tolerance toward diverse functional groups and structurally complex aromatic systems, underscores its potential as a practical, sustainable, and environmentally benign catalytic platform for the synthesis of aromatic amines.

### 3.3 Investigation of hydride sources

Nitrobenzene was initially selected as the model substrate for evaluating the catalytic performance of the AtPdNP catalyst. However, because nitrobenzene does not contain additional reducible functional groups, it was unsuitable for assessing the chemoselectivity of different hydrogen donors. To better investigate the functional-group tolerance and selectivity of the catalytic system, substrates bearing both nitro and carbonyl functionalities were considered. Accordingly, 4-nitroacetophenone was chosen as the model substrate due to the presence of both a reducible nitro group and a ketone moiety. To explore alternative hydrogen sources and their influence on product selectivity, ammonia borane (NH_3_BH_3_), hydrazine hydrate (NH_2_NH_2_), and molecular hydrogen (H_2_) were evaluated and compared with the NaBH_4_-based reduction system. The reduction outcomes obtained using these hydrogen donors are summarized in Scheme 3. The borane–ammonia complex exhibited limited chemoselectivity, resulting in the formation of a significant amount of the over-reduced product, 4-(1-hydroxyethyl)aniline. Approximately 50% of the product mixture consisted of this alcohol derivative, indicating simultaneous reduction of both the nitro and ketone functionalities. A similar but more pronounced effect was observed when hydrazine was employed as the reducing agent.

**Scheme 3:**
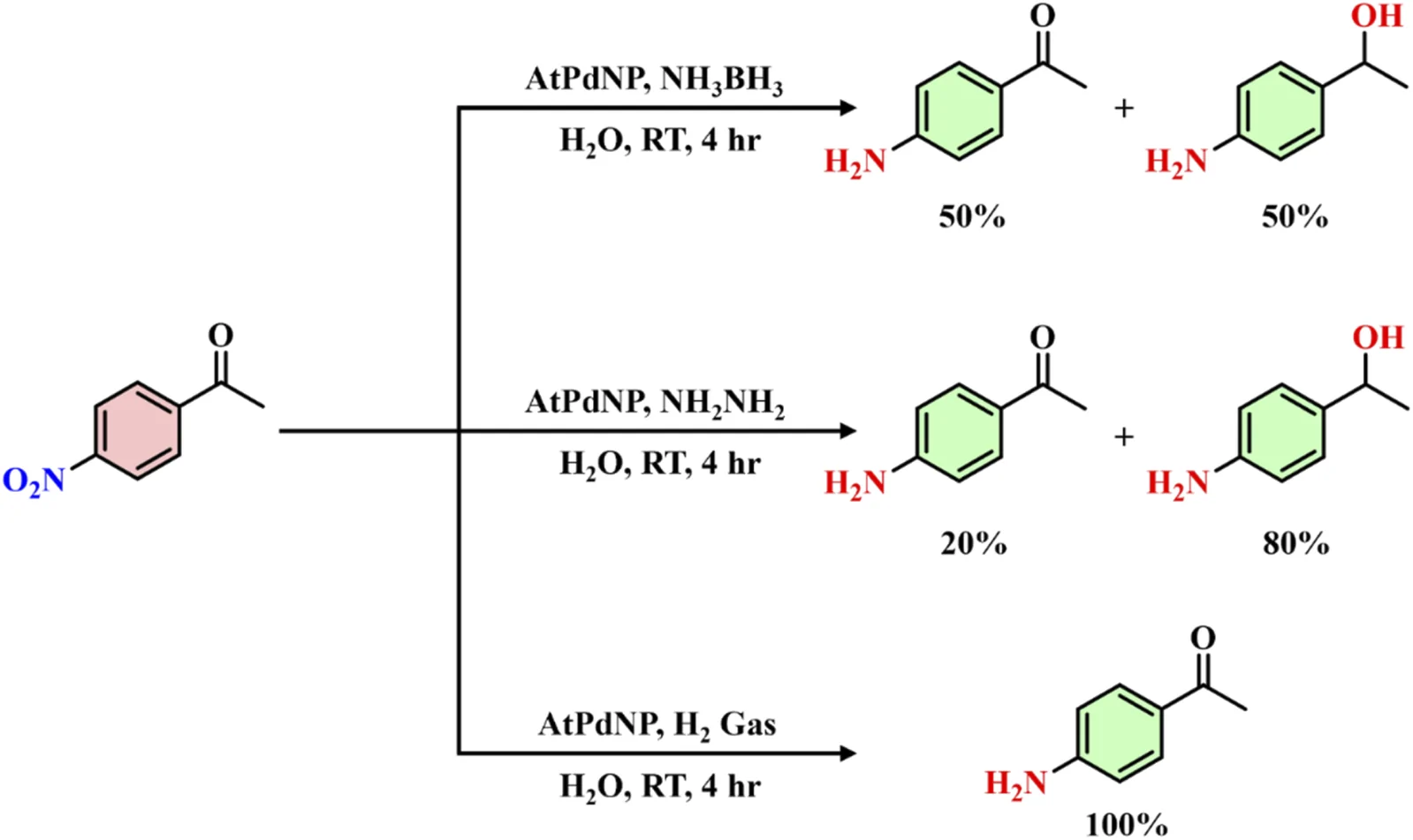
Change in the hydride source in nitro reduction (NH_3_BH_3_, NH_2_NH_2_, and H_2_).

In this case, the over-reduced alcohol product was formed preferentially, accounting for approximately 80% of the isolated products. In contrast, the use of molecular hydrogen (H_2_) under gas-purging conditions demonstrated superior chemoselectivity. The reduction proceeded predominantly through selective hydrogenation of the nitro group while preserving the ketone functionality, affording 4-aminoacetophenone as the major product. The formation of the corresponding alcohol derivative was effectively suppressed, highlighting the excellent selectivity of H_2_ as a hydrogen source in combination with the AtPdNP catalyst. These results demonstrate that the nature of the hydride or hydrogen donor plays a crucial role in determining product selectivity. While ammonia borane and hydrazine promote concurrent reduction of nitro and carbonyl groups, molecular hydrogen enables highly selective nitro-group reduction, making it a preferred reducing agent when preservation of sensitive carbonyl functionalities is desired.

### 3.4 Reaction under dark conditions

To evaluate the influence of light on the catalytic reduction process and to assess the suitability of the AtPdNP catalyst for industrial-scale applications, a control experiment was conducted under completely dark conditions. This investigation was particularly important because large-scale industrial reactors are typically opaque, limiting or entirely preventing exposure of the reaction mixture to light. The reduction reaction was performed under the optimized conditions while excluding all external light sources (Scheme 4). A comparison of the results obtained under dark and ambient laboratory conditions revealed no significant differences in reaction rate, substrate conversion, or isolated product yield. Complete conversion of the nitro substrate and comparable product yields were achieved in both cases. The absence of any observable light-dependent effect indicates that the catalytic reduction proceeds through a non-photochemical pathway.

**Scheme 4:**
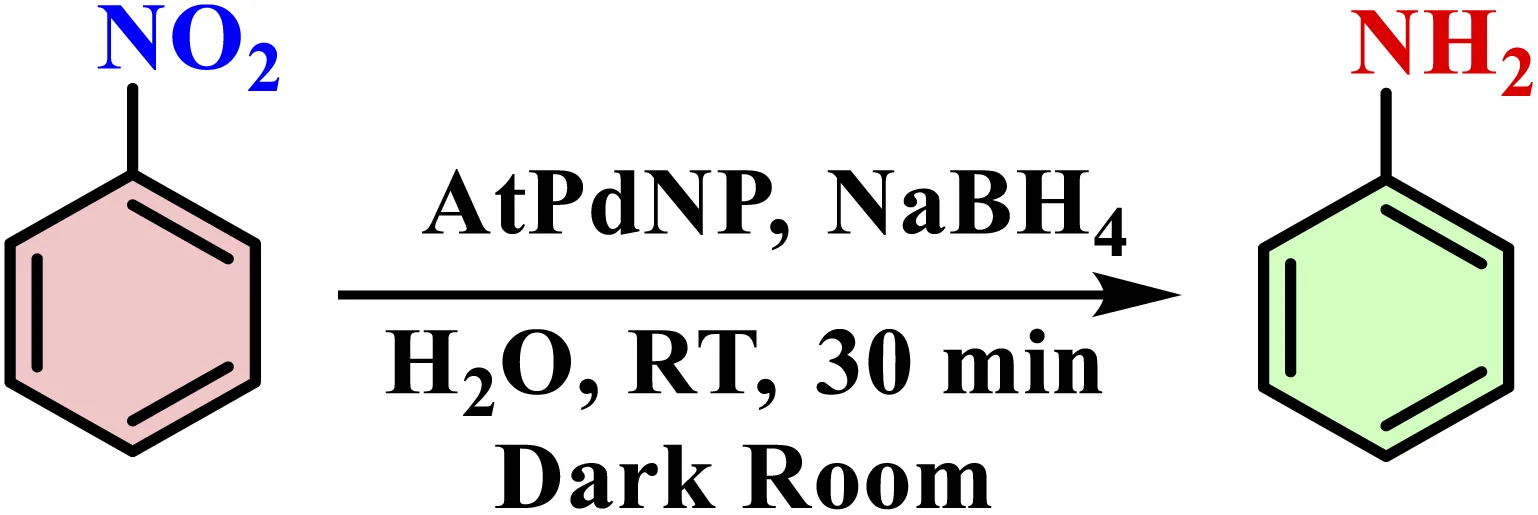
Nitro reduction under dark conditions.

Therefore, the AtPdNP-catalyzed reduction is driven primarily by thermal and surface-catalyzed processes rather than by photo-induced electron-transfer mechanisms. These findings confirm the robustness of the catalytic system and support its practical applicability for large-scale industrial operations, where light exposure is typically minimal or absent.

### 3.5 Investigation of the mechanism associated with the AtPdNP catalyst

The catalytic reduction of nitroaromatic compounds with metal nanoparticles has been reported in the literature to follow either of two different mechanistic pathways: the direct hydrogenation pathway or the indirect condensation pathway. In the direct pathway, stepwise/interfacial reductions occur successively on the nitro compound to give monomeric intermediates such as nitrosobenzene and phenylhydroxylamine, which are finally reduced to the corresponding anilines. In contrast, the indirect pathway proceeds through bimolecular condensation of these highly reactive primary intermediates (nitrosobenzene and phenylhydroxylamine) to form nitrogen–nitrogen (N–N) coupled species. This cascade gives rise to azoxybenzene, which is deoxygenated to azobenzene and subsequently reduced through hydrazobenzene (1,2-diphenylhydrazine) to the ultimate primary amine.^7–9,22,28^

To understand the precise reaction pathway guided by the synthesized AtPdNP catalyst, a time-course mechanistic probe was performed. Aliquots were directly withdrawn from the reaction mixture after 15 minutes, appropriately diluted/filtered, and analysed without isolation or purification. Therefore, the HPLC and LC-MS chromatograms of the intermediate-stage reaction mixture and the final reaction mixture contain the corresponding reaction components and associated impurities. These HPLC and LC-MS data were intended primarily for reaction monitoring with respect to the formation of various intermediates. The obtained mass spectra (Fig. S1 and S2) clearly indicated the existence of N–N coupled intermediates. The analysis showed characteristic signals at *m*/*z* = 199.3 and *m*/*z* = 183.4. These signals are fully consistent with the protonated molecular ions ([M + H]^+^) of 1,2-diphenyldiazene 1-oxide (azoxybenzene) and azobenzene, respectively.

Further, NMR characterization confirmed diphenyldiazene 1-oxide (azoxybenzene) as one selective intermediate. The sample was withdrawn from the reaction mixture after approximately 15 min, followed by appropriate work-up and purification before spectroscopic analysis. Thus, the clean NMR spectrum (Fig. S4 and S5) represents the worked-up/purified intermediate sample and not the crude reaction mixture.

The clear detection of these particular bimolecular condensation products confirms that the reduction catalysed by AtPdNP is not only *via* simple direct hydrogenation. In particular, the isolated azoxy intermediates strongly suggest that the reaction proceeds *via* the indirect condensation pathway (Scheme 5, Condensation Route), supporting the proposed complete mechanistic pathway of AtPdNP-catalyzed reduction of nitro compounds.

**Scheme 5:**
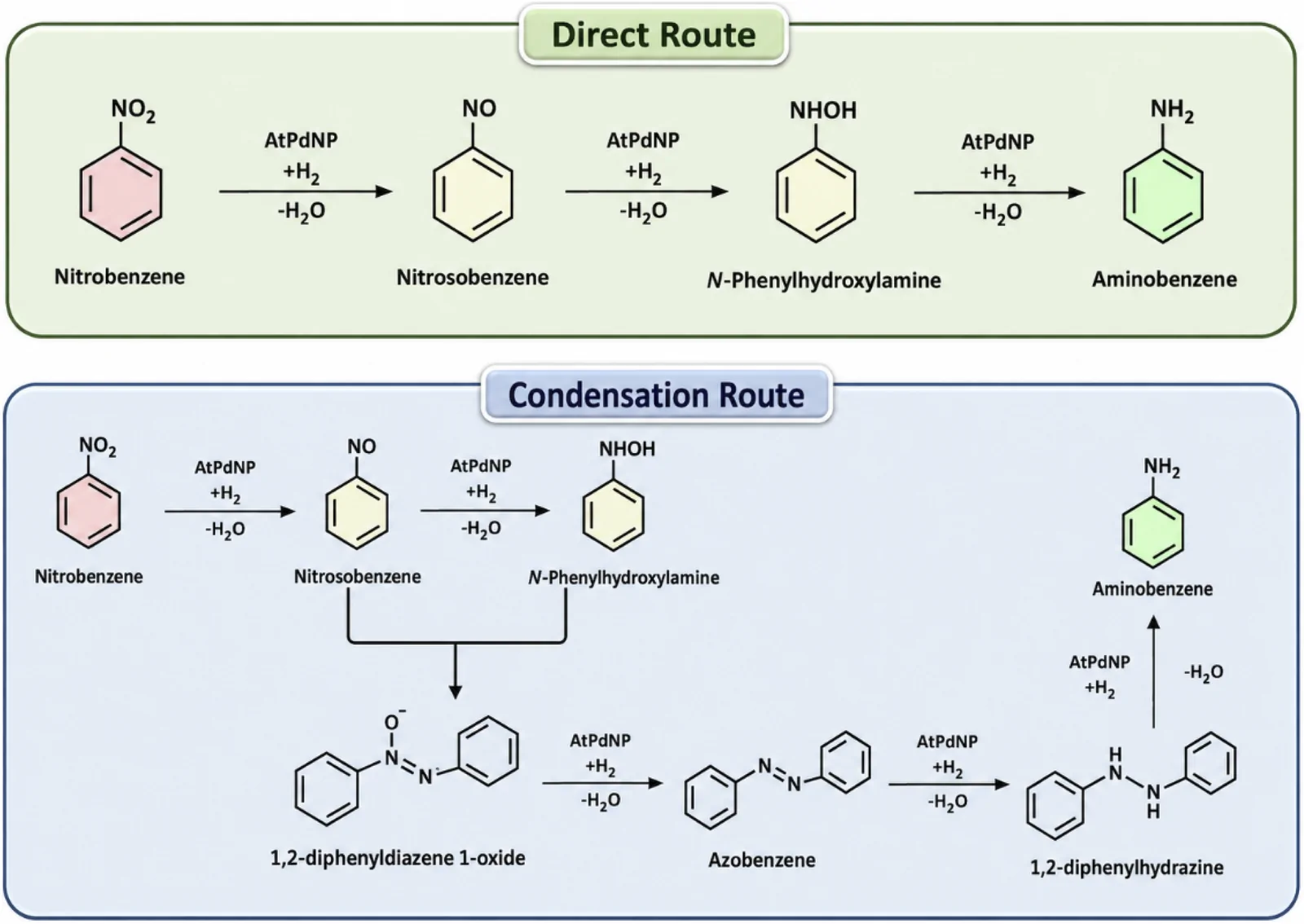
Investigating mechanism of nitrobenzene reduction using AtPdNP.

### 3.6 Reusability and operational stability of the AtPdNP catalyst

#### Reusability study

The practical applicability of a heterogeneous catalyst largely depends on its reusability, operational stability, and retention of catalytic activity over multiple reaction cycles. To evaluate these characteristics, the reusability of the AtPdNP catalyst was investigated using the reduction of nitroarenes to the corresponding anilines as a model reaction. As illustrated in Fig. 1 and Table S3, the AtPdNP catalyst exhibited excellent durability and sustained catalytic performance over ten consecutive reaction cycles. The catalyst afforded nearly complete conversion (∼100%) during the first cycle and showed only a marginal decline in activity upon successive reuse. Notably, substrate conversion remained above 90% even after the tenth catalytic cycle, demonstrating the remarkable stability of the biogenic palladium nanocatalyst under the reaction conditions. In addition, each reuse experiment was performed in triplicate, and the resulting low standard deviation values indicate excellent reproducibility and consistency of the catalytic performance. The minimal loss in activity observed during repeated use may be attributed to minor catalyst loss during recovery and handling or slight surface deactivation over extended operation. Nevertheless, the overall catalytic efficiency remained exceptionally high throughout the reusability study. To study the morphology of AtPdNP after the 10th cycle, the recovered catalyst was subjected to SEM (Fig. S9 and S10) and FT-IR (Fig. S6 and S8) characterization and compared with the corresponding analyses of the fresh catalyst. The SEM results indicate that the overall morphology of the catalyst was retained after repeated catalytic cycles, while the FT-IR spectrum of the recovered catalyst retained the characteristic vibrational features observed for the fresh AtPdNP catalyst. These results support the preservation of the catalyst's morphological and chemical characteristics after repeated use. Furthermore, ICP analysis was performed to quantitatively investigate Pd leaching. The reaction mixture and the filtrate obtained after catalyst separation were analysed for Pd content. The ICP results (Table S6) revealed only a negligible amount of Pd in the analysed solution, demonstrating that significant Pd leaching does not occur under the employed reaction conditions. These studies highlight the robust nature of the AtPdNP catalyst and underscore its potential for practical and sustainable applications in the reduction of nitroarenes. The combination of high catalytic activity, excellent reusability, and operational stability makes AtPdNP a promising heterogeneous catalyst for environmentally benign and economically viable industrial processes.

**Fig. 1:**
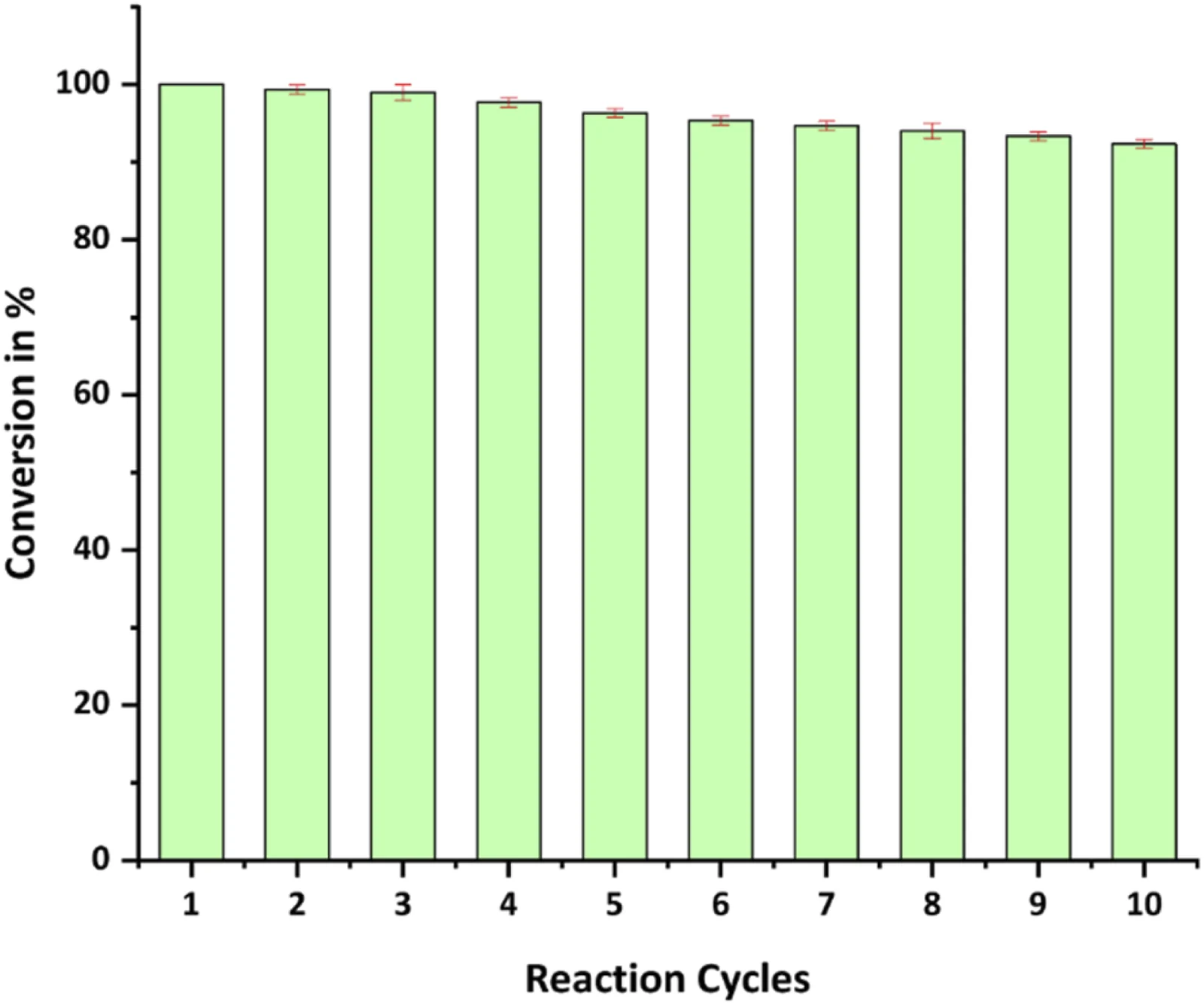
Reusability of AtPdNP in nitro reduction.

#### Storage study

The practical utility of a heterogeneous catalyst depends not only on its catalytic activity and reusability but also on its long-term storage stability. To assess the shelf life and durability of the AtPdNP catalyst, a longitudinal storage study was conducted over a period of 12 months. The catalyst was stored under ambient conditions in a hermetically sealed container to minimize exposure to atmospheric moisture, contaminants, and oxidizing agents. Samples were withdrawn at regular monthly intervals and evaluated for their catalytic performance in the reduction of nitroarenes under the optimized reaction conditions. Throughout the one-year study, the AtPdNP catalyst retained its catalytic activity and consistently afforded high substrate conversions, demonstrating excellent long-term stability. No significant decline in reduction efficiency was observed during the storage period, indicating that the catalyst preserved its active surface sites and structural integrity under ambient storage conditions. The remarkable stability of AtPdNP suggests strong resistance to common degradation pathways encountered in supported metal nanoparticle catalysts, including Ostwald ripening, nanoparticle agglomeration, particle growth, and structural collapse. Furthermore, the sustained catalytic performance indicates that the palladium active sites remain largely unaffected by spontaneous surface oxidation or catalyst poisoning during storage. The effectiveness of the hermetic storage conditions in preserving catalyst activity further supports the robustness of the biogenic nanocatalyst. These findings demonstrate that AtPdNP possesses excellent shelf life and operational readiness, making it a reliable catalyst for long-term storage and practical deployment. The combination of prolonged stability, high catalytic activity, and reusability enhances its attractiveness for industrial and large-scale applications where catalyst storage and transportation are important considerations (Fig. 2).

**Fig. 2:**
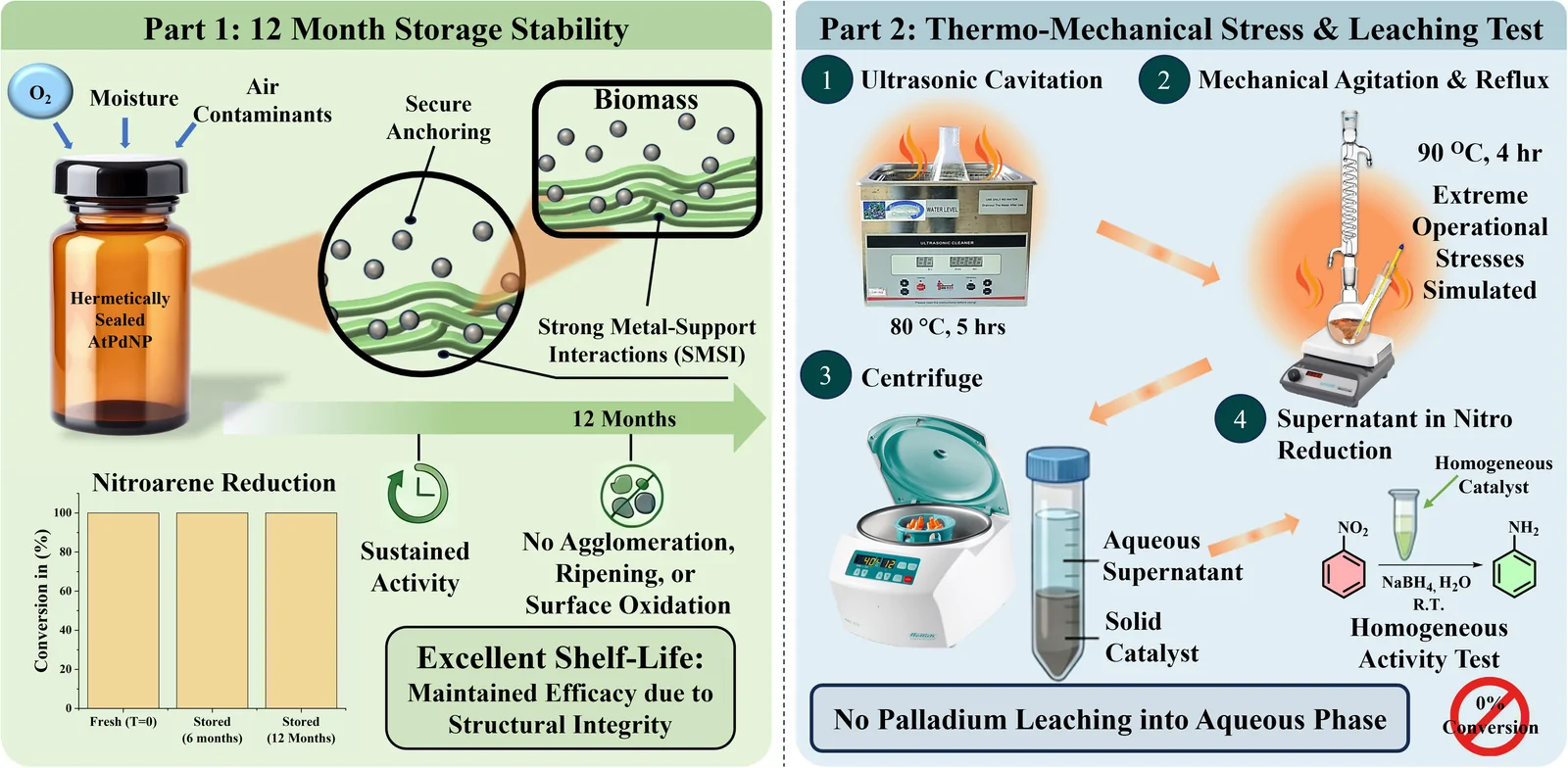
Storage and leaching study.

#### Leaching study

The susceptibility of the AtPdNP catalyst to active-phase leaching was investigated through a thermo-mechanical stability test designed to simulate severe operational conditions and evaluate the strength of palladium immobilization on the biomass support. In this study, the catalyst was dispersed in an aqueous medium and subjected to intensive ultrasonication at 80 °C for 5 h, followed by vigorous mechanical stirring under reflux at 90 °C for an additional 4 h. These harsh treatment conditions were intentionally selected to maximize the likelihood of nanoparticle detachment and metal leaching from the support matrix. After completion of the treatment, the solid catalyst was separated by centrifugation, and the resulting supernatant was collected and tested for catalytic activity using the reduction of nitrobenzene as a model reaction. No detectable conversion of nitrobenzene was observed in the presence of the supernatant alone, indicating the absence of catalytically active palladium species in solution. The lack of product formation demonstrates that palladium leaching, if any, was below the detectable limit of the catalytic assay and that the active palladium nanoparticles remained firmly associated with the biomass support even under severe thermo-mechanical stress. To further support this, we performed ICP analysis of the separated supernatant (Table S6) and FT-IR analysis of the catalyst. The FT-IR spectrum of the recovered catalyst (Fig. S7) retains characteristic vibrational features observed for the fresh AtPdNP catalyst (Fig. S6). The ICP results showed no detectable Pd in the supernatant within the detection limit of the analytical method, thereby providing direct quantitative evidence supporting the stability of Pd within the AtPdNP matrix under the applied reaction conditions. These findings suggest the existence of strong interactions between the palladium nanoparticles and the fungal biomass matrix, which effectively stabilize the active phase and prevent metal loss during operation.

The excellent resistance to leaching observed in this study highlights the heterogeneous nature of the AtPdNP catalyst and underscores its robustness for repeated use and long-term applications. The strong anchoring of palladium nanoparticles contributes significantly to catalyst durability, minimizes metal contamination of the reaction products, and enhances the sustainability and economic viability of the catalytic system (Fig. 2).

## 4 Conclusion

In summary, a biogenic palladium nanocatalyst (AtPdNP) synthesized using *Aspergillus trinidadensis* VM ST01′ was successfully employed as an efficient heterogeneous catalyst for the reduction of nitroarenes to the corresponding amines under mild and environmentally benign conditions. The catalytic system utilizes sodium borohydride as a hydrogen donor in aqueous medium at room temperature, providing a simple, sustainable, and operationally convenient approach for the synthesis of aromatic amines. Optimization studies established the ideal reaction parameters, enabling rapid and highly efficient reduction of nitrobenzene and a broad range of substituted nitroarenes. The catalyst demonstrated excellent activity toward substrates bearing electron-donating and electron-withdrawing groups, including halogens, hydroxyl, amino, alkyl, and heterocyclic functionalities. High conversions and isolated yields were achieved within short reaction times, highlighting the remarkable catalytic efficiency of the AtPdNP system. Furthermore, the catalyst exhibited excellent chemoselectivity, preserving sensitive functional groups such as aryl halides while effectively reducing nitro functionalities. The catalytic system also proved effective for the reduction of sterically demanding bicyclic and polycyclic nitroaromatic compounds, demonstrating broad substrate compatibility. Mechanistic investigations confirmed that the reduction proceeds through a thermal catalytic pathway, as complete exclusion of light had no influence on reaction performance. Comparative studies with alternative hydrogen donors revealed that the nature of the reducing agent significantly affects chemoselectivity, with sodium borohydride providing an effective balance between reactivity and substrate scope under the optimized conditions. From a practical standpoint, the AtPdNP catalyst exhibited outstanding reusability, maintaining high catalytic activity over ten consecutive reaction cycles with only minimal loss in performance. In addition, the catalyst retained its activity during a 12-month storage study under ambient conditions, demonstrating excellent shelf life and long-term stability. Thermo-mechanical stress testing further confirmed the strong immobilization of palladium nanoparticles on the fungal biomass support, with no detectable catalytic activity observed in the separated supernatant after severe treatment, indicating excellent resistance to active-phase leaching. Overall, the AtPdNP catalyst combines high catalytic efficiency, broad substrate scope, excellent stability, reusability, and environmentally friendly reaction conditions. These attributes make it a promising and sustainable catalytic platform for the selective reduction of nitroarenes and highlight its potential for practical and large-scale applications in fine chemical, pharmaceutical, and specialty chemical synthesis.

## Author contributions

All the experiments were designed and performed by Hemal B. Rathod and Paresh N. Patel. The spectral study, interpretation, and correlations were done by Hemal B. Rathod, Yashasvi N. Desai and Paresh N. Patel. The manuscript was written by Hemal B. Rathod. The final proofreading and editing were done by Paresh N. Patel.

## Conflicts of interest

The authors declare that they have no known competing financial interests or personal relationships that could have appeared to influence the work reported in this paper.

## Supplementary Material

NA-OLF-D6NA00568C-s001

## Data Availability

Additional data related to the main manuscript are available in the supplementary information (SI). Supplementary information is available. See DOI: https://doi.org/10.1039/d6na00568c.
