# Supplementary material for: Evaluation of a highly stable biogenic palladium nanocatalyst for industrially relevant and sustainable nitro reduction
Source: Nanoscale Adv. 2026 Sep 28;8(22):6808–19. doi: 10.1039/d6na00568c (PMC13670218; doi:10.1039/d6na00568c)
Supplement: Supplementary file 1 [file NA-008-D6NA00568C-s001.pdf]

Supplementary Information (SI) for Nanoscale Advances.
This journal is © The Royal Society of Chemistry 2026
Supplementary Information (SI) for Nanoscale Advances.
This journal is © The Royal Society of Chemistry

Evaluation of a Highly Stable Biogenic Palladium Nanocatalyst for Industrially Relevant and Sustainable Nitro Reduction

Hemal B. Rathod, Yashasvi N. Desai, Paresh N. Patel*

Laboratory of Bio-Organic Chemistry, Tarsadia Institute of Chemical Science (TICS), Uka Tarsadia University, Bardoli – 394350, Gujarat, India

*e-mail: pareshn111@yahoo.com

Supplementary

1 Investigation of the mechanism associated with the AtPdNP catalyst

1.1 Mass Data

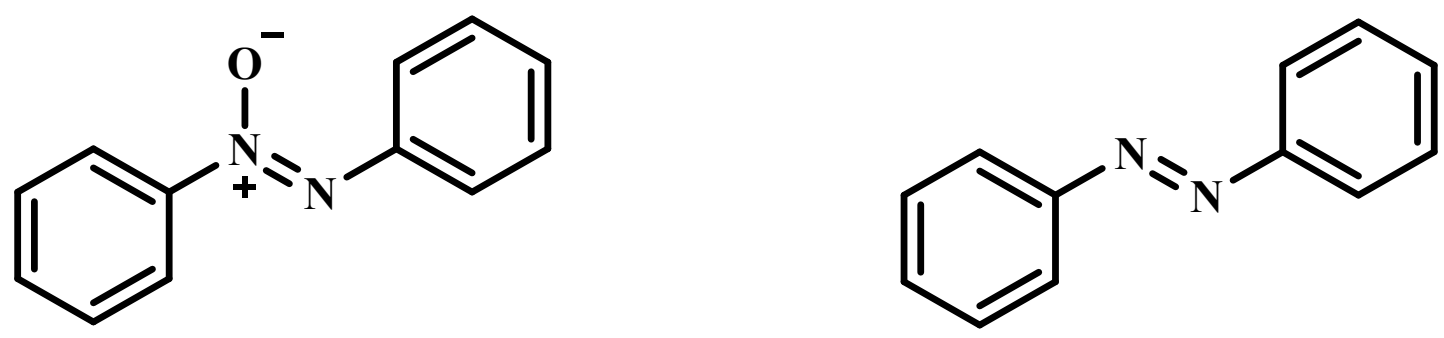

1,2-diphenyldiazene 1-oxide
m/z: 198.08 (100.0%), 199.08 (13.7%)

Azobenzene
m/z: 182.08 (100.0%), 183.09 (13.1%)

Scheme S1: Structure and mass of the stable intermediate

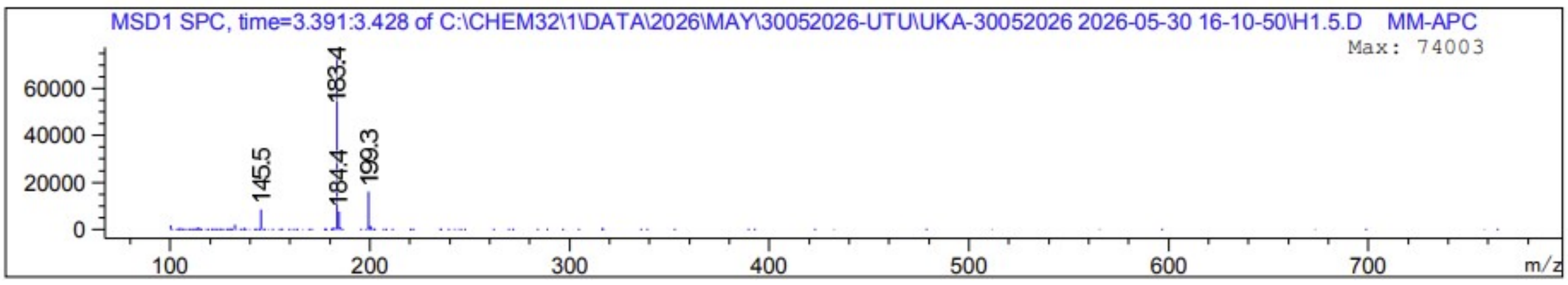

Figure S1: LC-MS mass Chromatogram of intermediate

1.2 HPLC data

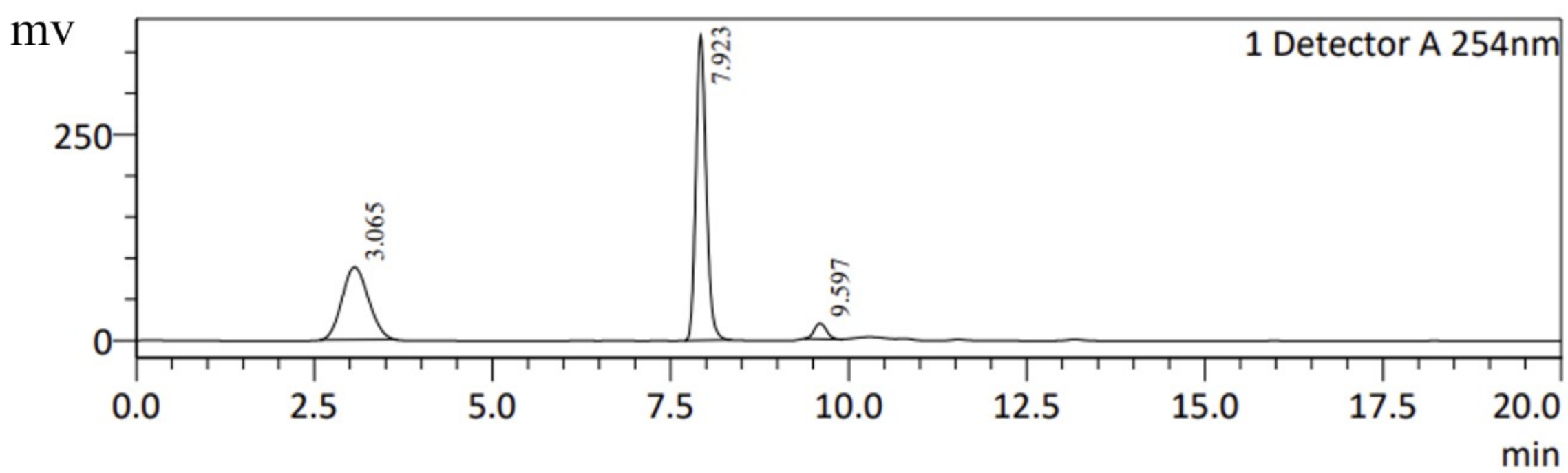

Figure S2: HPLC Chromatogram of intermediate

Table S1: HPLC Peak Table

Name	Peak#	Ret. Time	Area	Area%
Intermediate	1	3.065	2300199	36.751
Aniline	2	7.923	3732296	59.632
Impurity	3	9.597	226370	3.617
	**Total**		**6258865**	**100.000**

2 HPLC Data of Model Reaction

a) Standard Nitrobenzene

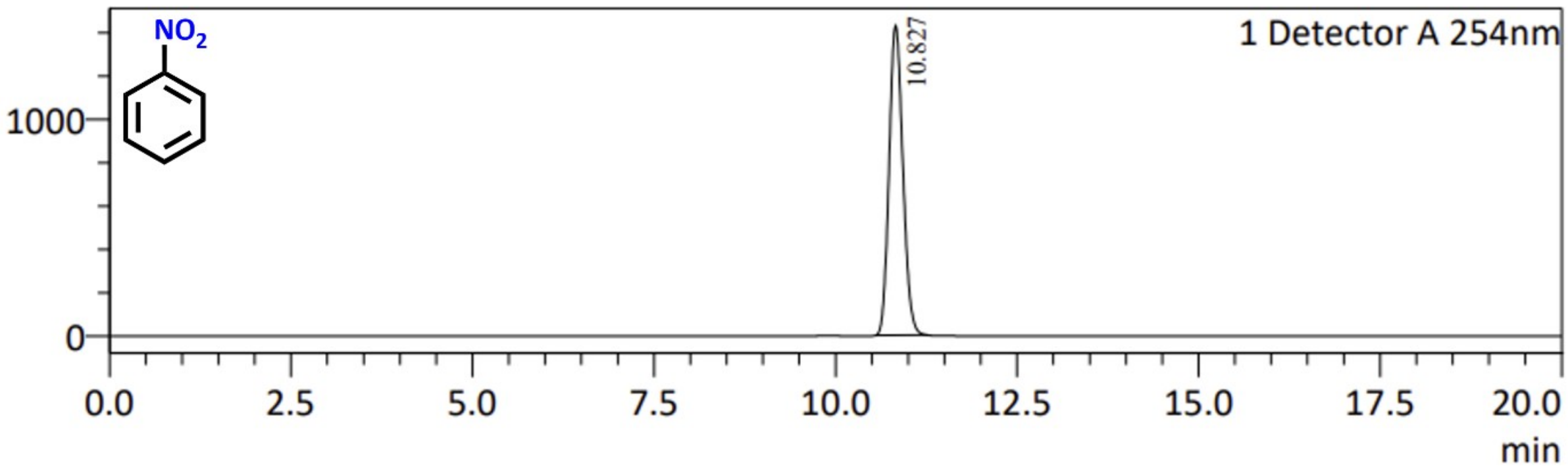

b) Standard Aniline

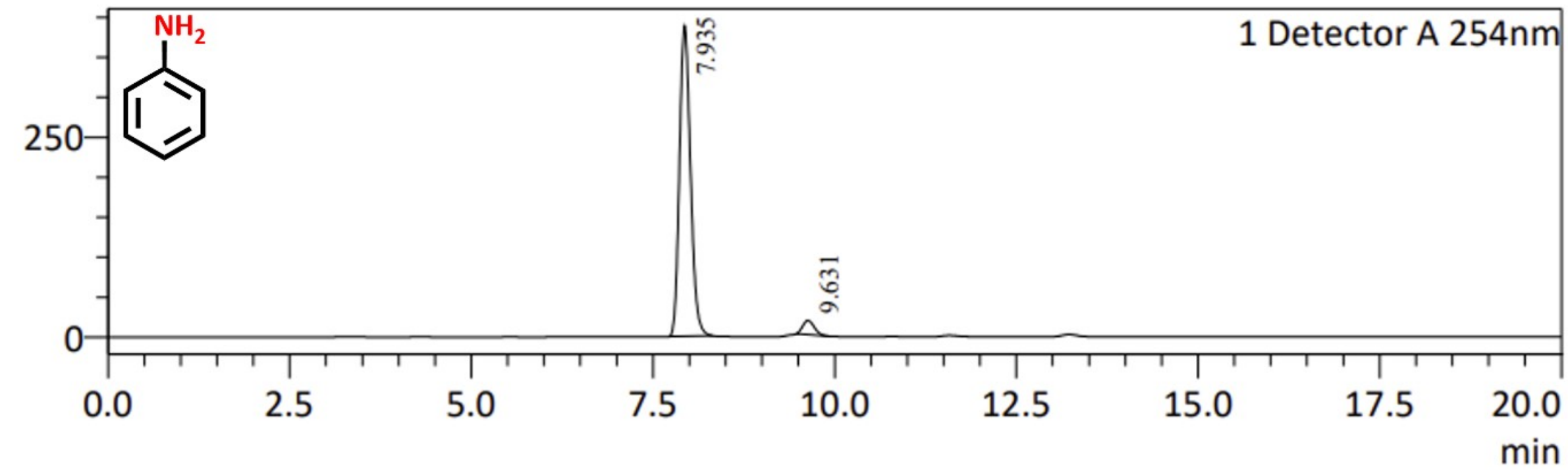

c) Nitrobenzene conversion into Aniline using AtPdNP catalyst

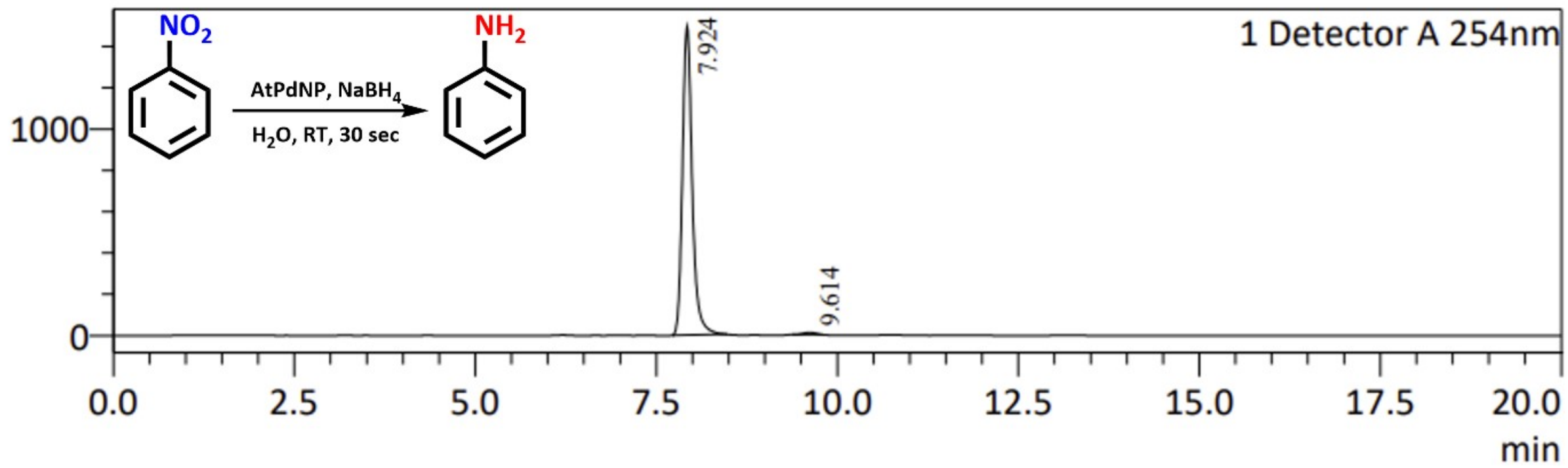

Figure S3: HPLC chromatogram of (Panel 1) standard reference nitrobenzene, (Panel 2) standard reference aniline, (Panel 3) synthesized aniline

Table S2: HPLC Peak Table

Name	Peak#	Ret. Time	Area	Area %
**(Panel 1) HPLC Peak Table for Standard Reference Nitrobenzene**				
Nitrobenzene	1	10.827	18871343	100.00
	**Total**		**18871343**	**100.00**
**(Panel 2) HPLC Peak Table for Standard Reference Aniline**				
Aniline	1	7.935	4090000	95.449
Impurity	2	9.631	194997	4.551
	**Total**		**4284997**	**100.00**
**(Panel 3) HPLC Peak Table for Synthesized Aniline**				
Aniline	1	7.886	2448607	96.746
Impurity	2	9.528	82351	3.254
	**Total**		**2530958**	**100.00**

3 NMR of Intermediate

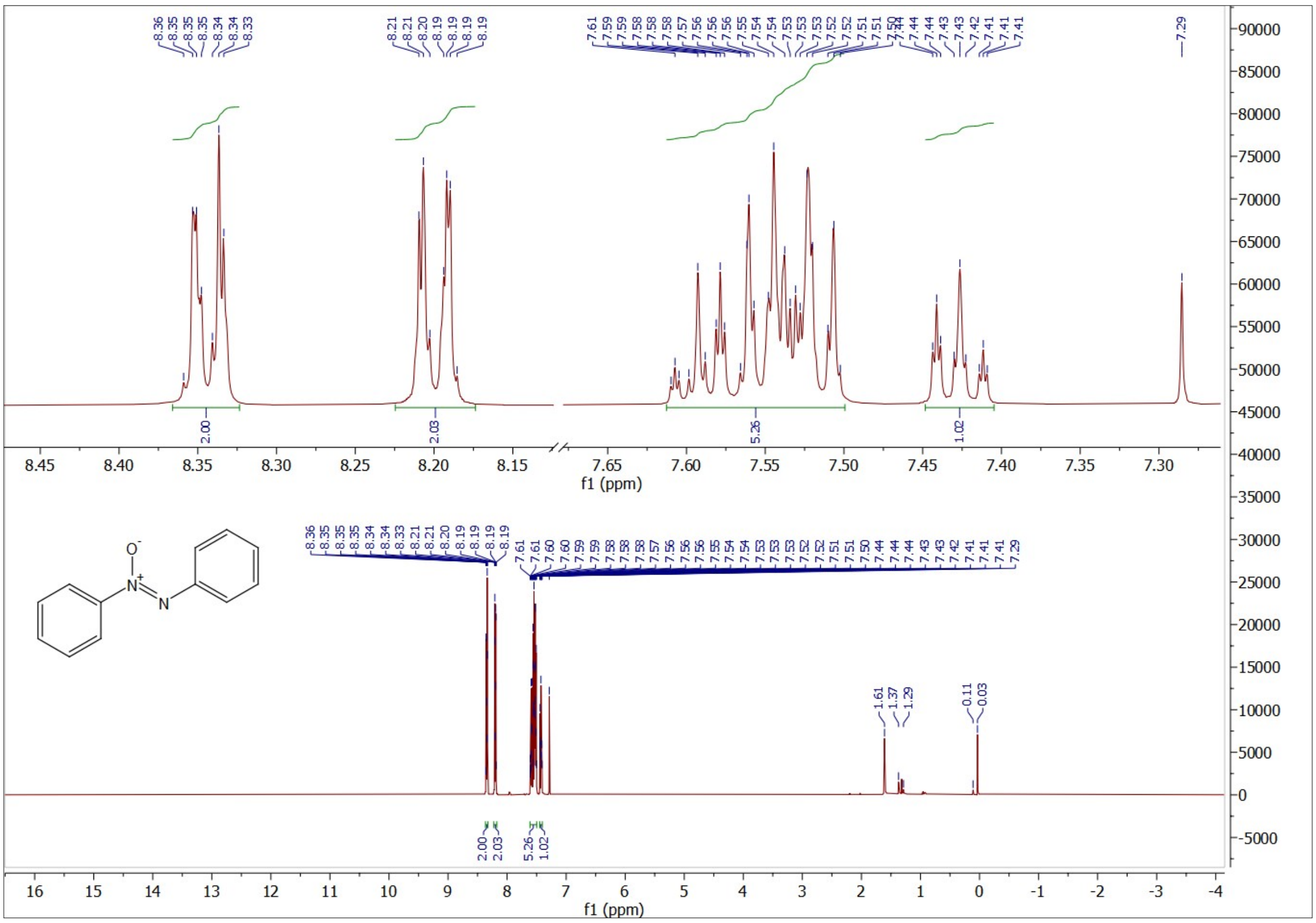

Figure S4: ^{1}H NMR Spectrum of intermediate 1,2-diphenyldiazene 1-oxide

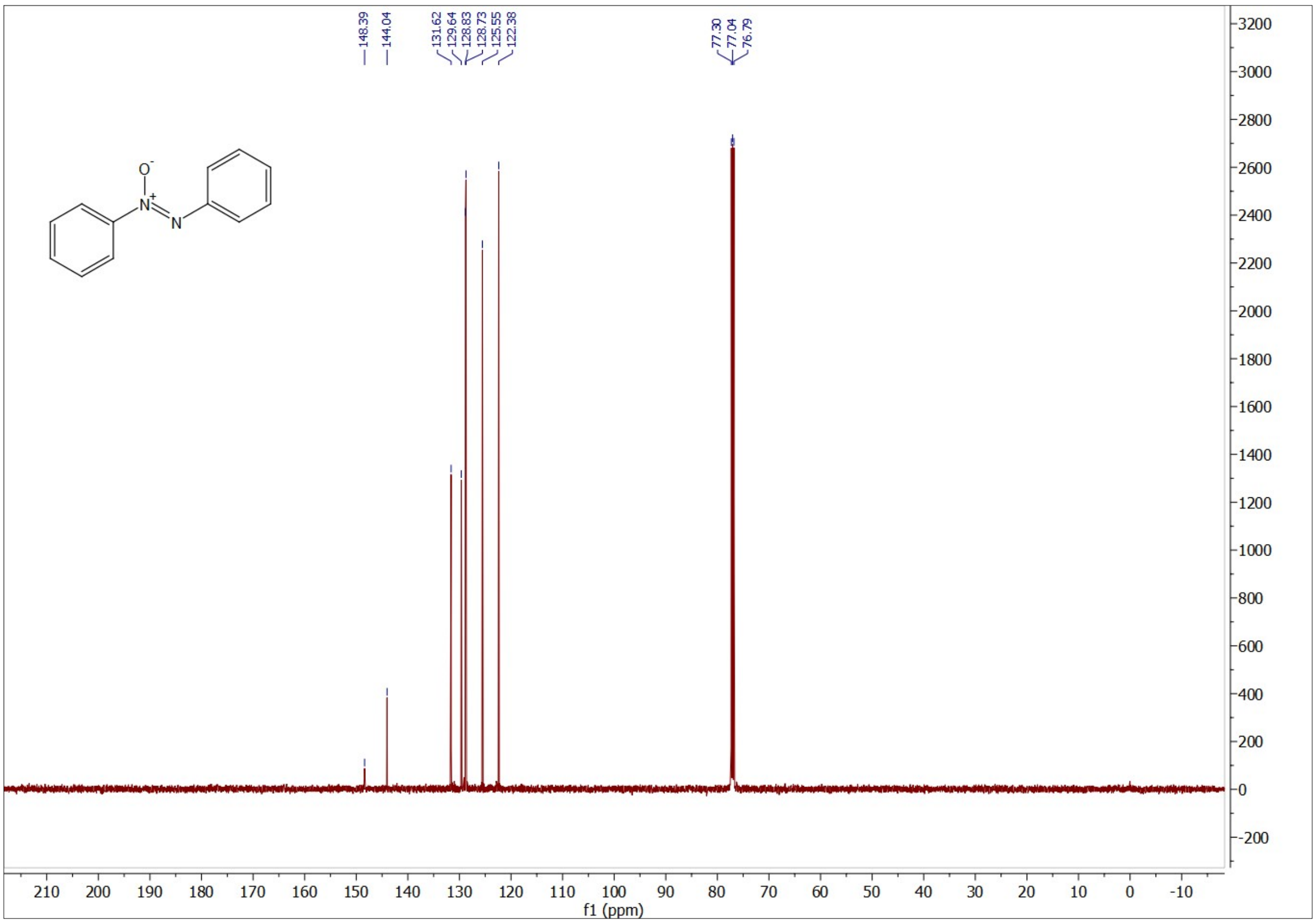

Figure S5: ^{13}C NMR Spectrum of intermediate 1,2-diphenyldiazene 1-oxide

4 AtPdNP Recycling Data

Table S3: Reusability data of triplicate experiments

Reaction Cycles	1st time Conversion (%)	2nd time Conversion (%)	3rd time Conversion (%)	Mean (%)	SD (%)
**1**	100	100	100	100.00	0.00
**2**	99	100	99	99.33	0.57
**3**	100	99	98	99.00	1.00
**4**	98	97	98	97.66	0.57
**5**	96	97	96	96.33	0.57
**6**	95	95	96	95.33	0.57
**7**	94	95	95	94.66	0.57
**8**	95	94	93	94.00	1.00
**9**	93	94	93	93.33	0.57
**10**	92	92	93	92.33	0.57

5 Yield Table for Table 3 and Table 4

Table S4: Yield data for substrates from table 3

Sr. No.	1st Yield %	2nd Yield %	3rd Yield %	Mean	Standard Deviation
1	95.30	94.60	95.70	95.20	0.55
2	96.00	95.60	95.02	95.54	0.49
3	94.10	92.04	93.15	93.09	1.03
4	93.89	95.78	95.40	95.02	0.99
5	94.25	95.70	95.30	95.08	0.74
6	91.50	90.25	90.40	90.71	0.68
7	92.30	93.10	91.60	92.33	0.75
8	94.10	96.20	95.42	95.24	1.06
9	92.25	92.46	91.36	92.02	0.58
10	95.34	95.87	93.80	95.00	1.07
11	95.48	96.24	95.10	95.60	0.58
12	96.25	95.45	94.62	95.44	0.81
13	94.80	95.90	94.36	95.02	0.79
14	85.75	84.20	86.10	85.35	1.01
15	95.25	95.36	95.24	95.28	0.06
16	95.60	94.95	95.15	95.23	0.33
17	96.58	94.57	95.10	95.41	1.04
18	-	-	-	-	-
19	97.11	95.24	95.17	95.84	1.10
20	95.65	95.17	95.75	95.52	0.31
21	94.62	95.90	95.38	95.30	0.64

Table S5: Yield data for substrates from table 4

Sr. No.	1st Yield %	2nd Yield %	3rd Yield %	Mean	Standard Deviation
1	81.24	80.60	80.35	80.73	0.45
2	94.25	95.80	95.42	95.15	0.80
3	79.95	80.42	80.30	80.22	0.24
4	90.63	90.74	89.65	90.34	0.60
5	95.43	95.61	94.75	95.26	0.45
6	96.25	94.65	95.40	95.43	0.80
7	94.25	95.80	95.43	95.16	0.80
8	95.16	95.86	94.95	95.32	0.47
9	85.89	84.65	84.95	85.16	0.64
10	94.38	95.61	95.25	95.08	0.63
11	95.34	95.16	95.02	95.17	0.16
12	95.26	94.87	94.95	95.02	0.20
13	84.65	85.43	85.28	85.12	0.41
14	85.26	85.42	85.03	85.23	0.19

6 Catalyst leaching study by ICP-OES

Table S6: Catalyst leaching study by ICP-OES

Sample Code	Pd in AtPdNP (PPM)	Pd Leaching (PPM)	Pd Leaching (% PPM)
**A**	530	3.15	0.59
**B**	530	5.99	1.13
**C**	530	6.03	1.13

**D**	530	5.92	1.11
**E**	530	3.73	0.70

The resultant A-catalyst solution was stirred for 24 h, B-catalyst solution was sonicated at 80 °C for 6 h, C-catalyst solution was used for nitro reduction with sodium borohydride for 1 h, D-after thermo-mechanical stress, and E-catalyst was used after 10 reaction cycles. Later, the catalyst was separated by filtration, and the filtrate solutions were used for ICP-OES analysis (Table S6).

7 FT-IR data for catalyst AtPdNP

- **FT-IR of Fresh Catalyst AtPdNP**

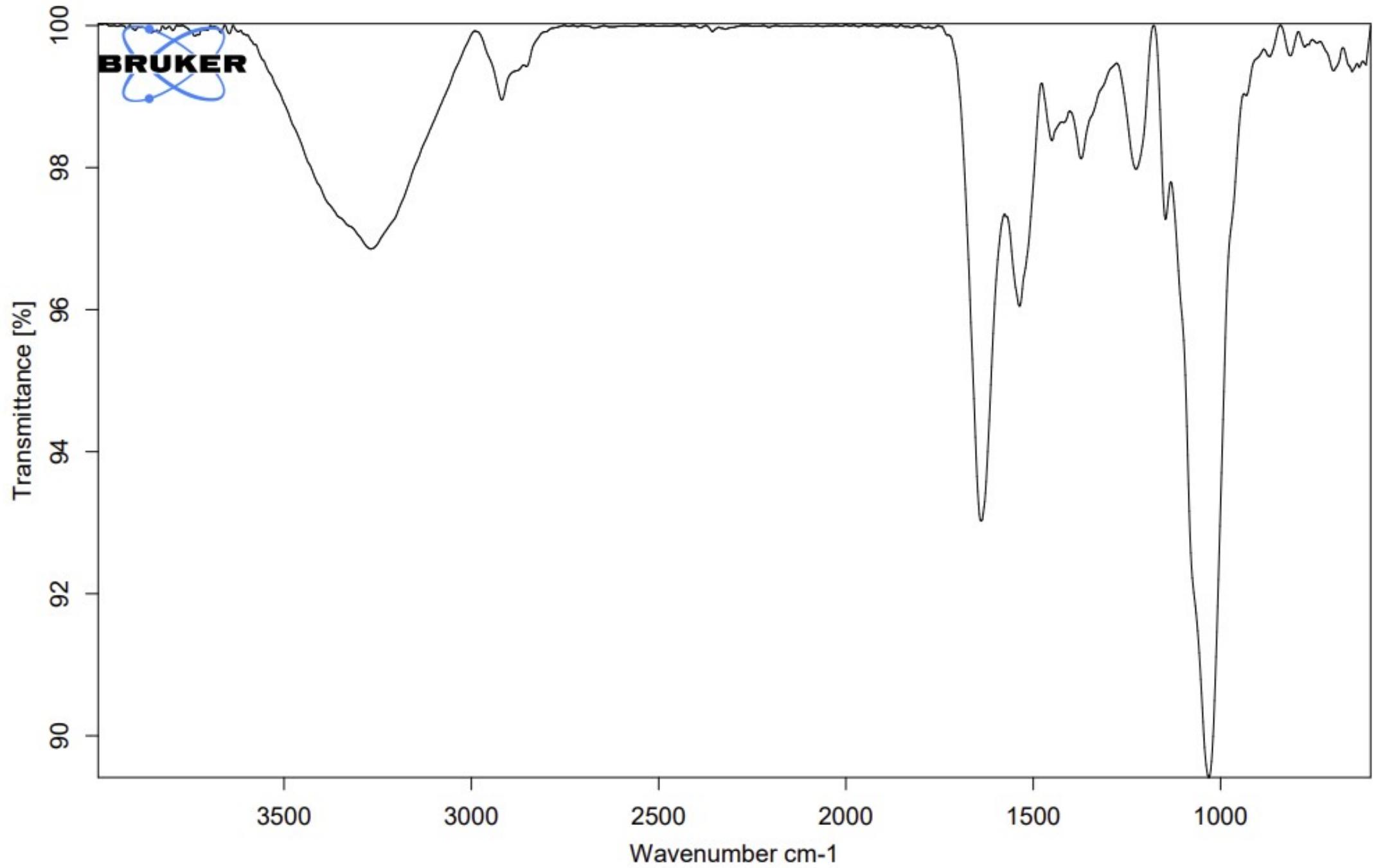

Figure S6: FT-IR of fresh AtPdNP

- **FT-IR of Catalyst AtPdNP After Thermomechanical Stress**

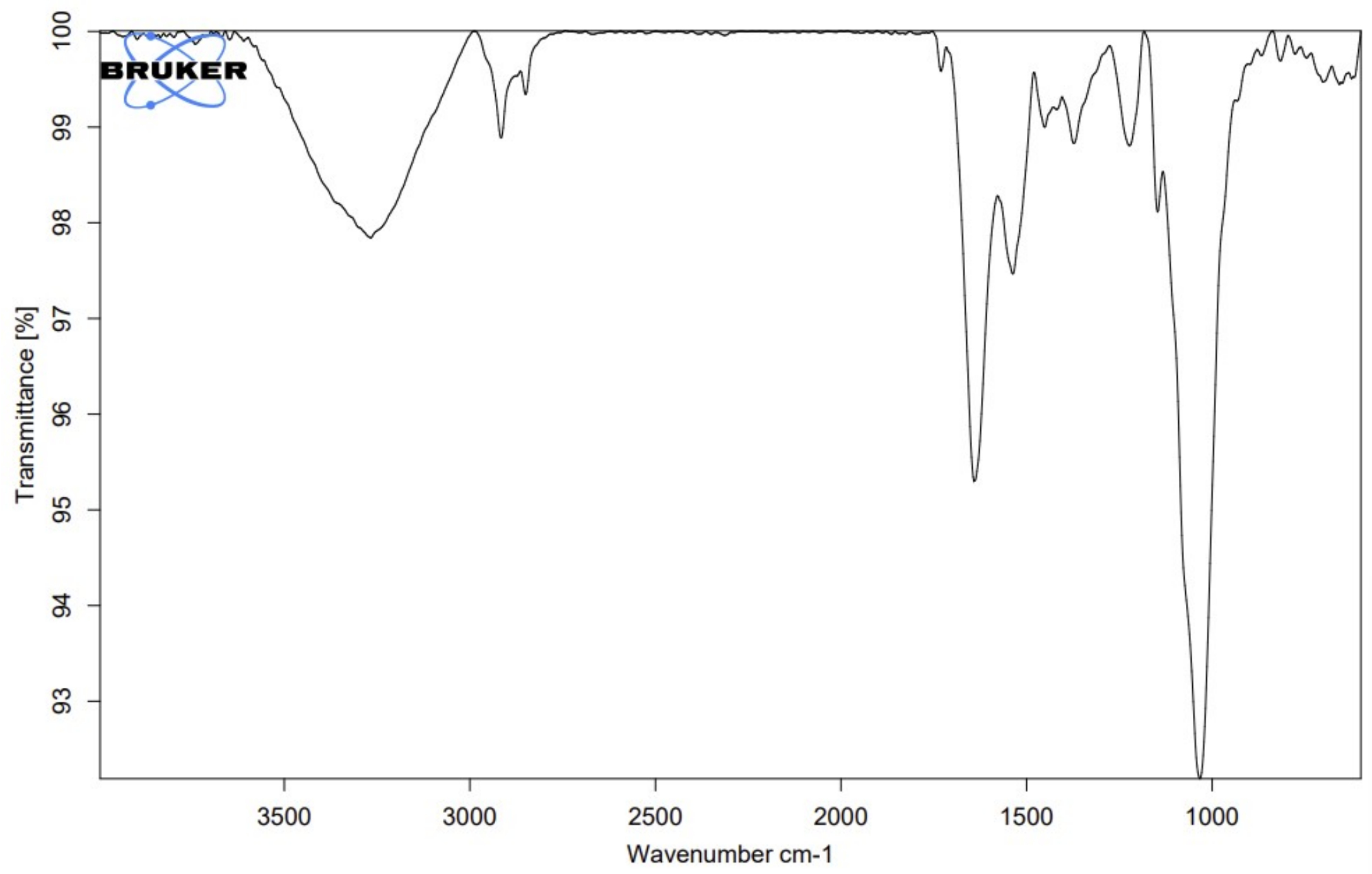

Figure S7: FT-IR of AtPdNP after thermomechanical stress

➢ **FT-IR of Catalyst AtPdNP After 10 Cycles of Reusability**

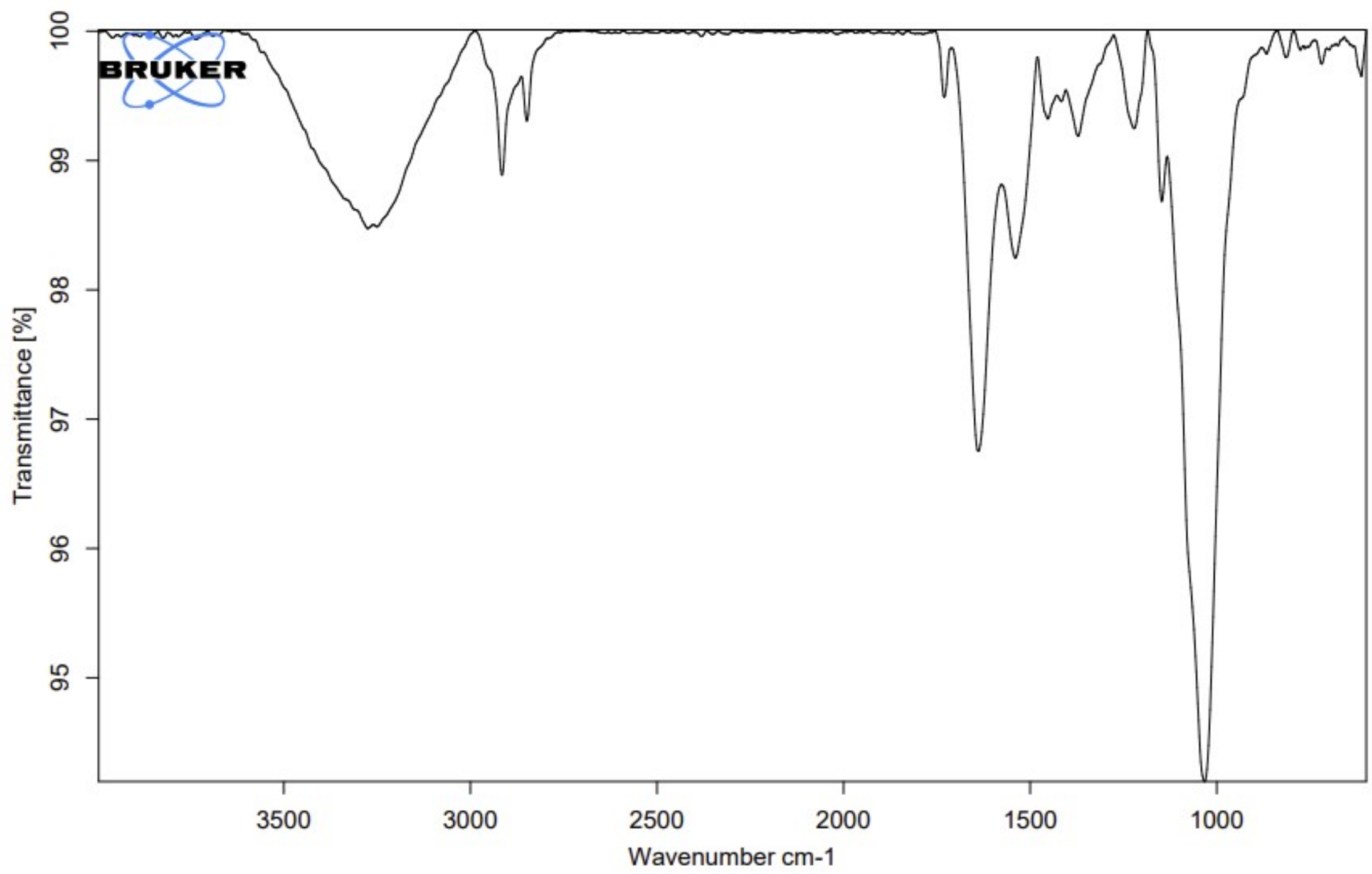

Figure S8: FT-IR of AtPdNP after 10 Cycles of Reusability

8 SEM Images of AtPdNP

➢ **SEM Image of Fresh Catalyst AtPdNP [1]**

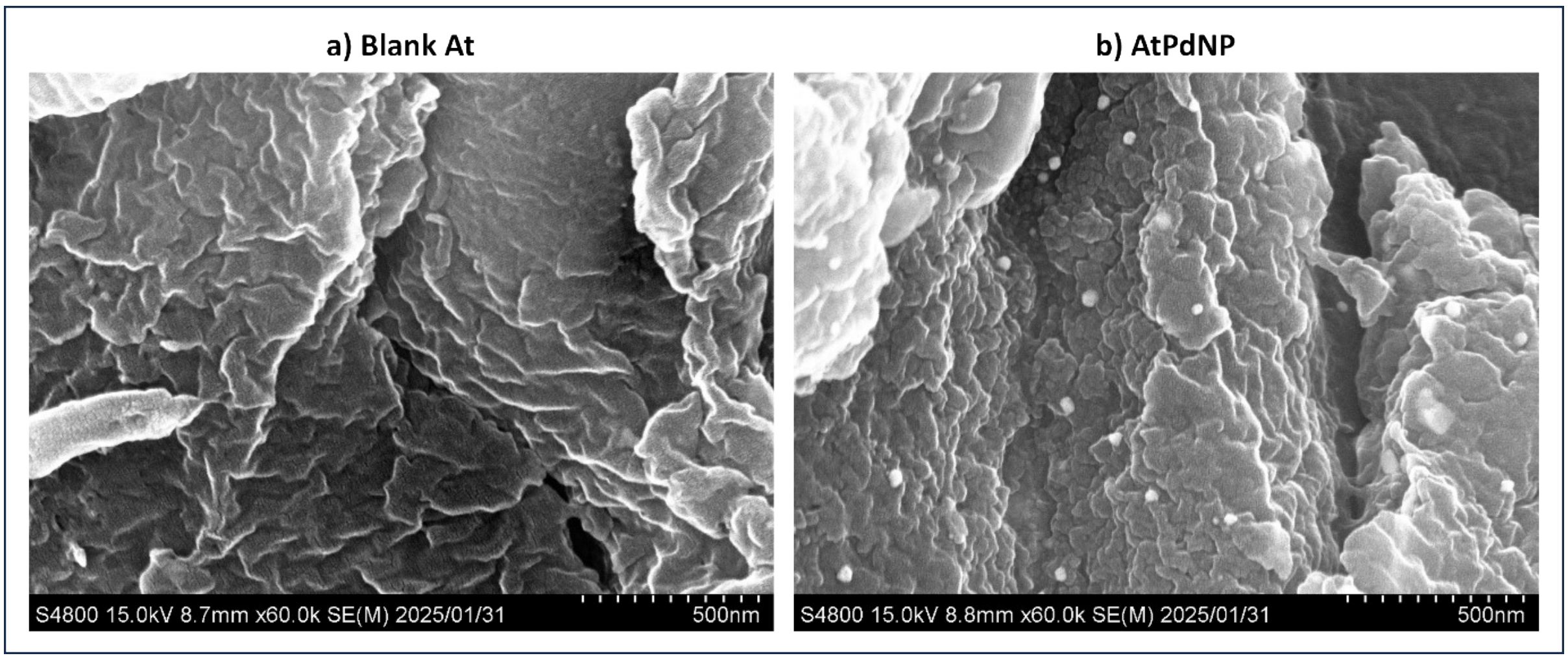

Figure S9: SEM images of (a) blank At and (b) AtPdNP catalyst

- **SEM Image of Catalyst AtPdNP After 10th cycle of reuse**

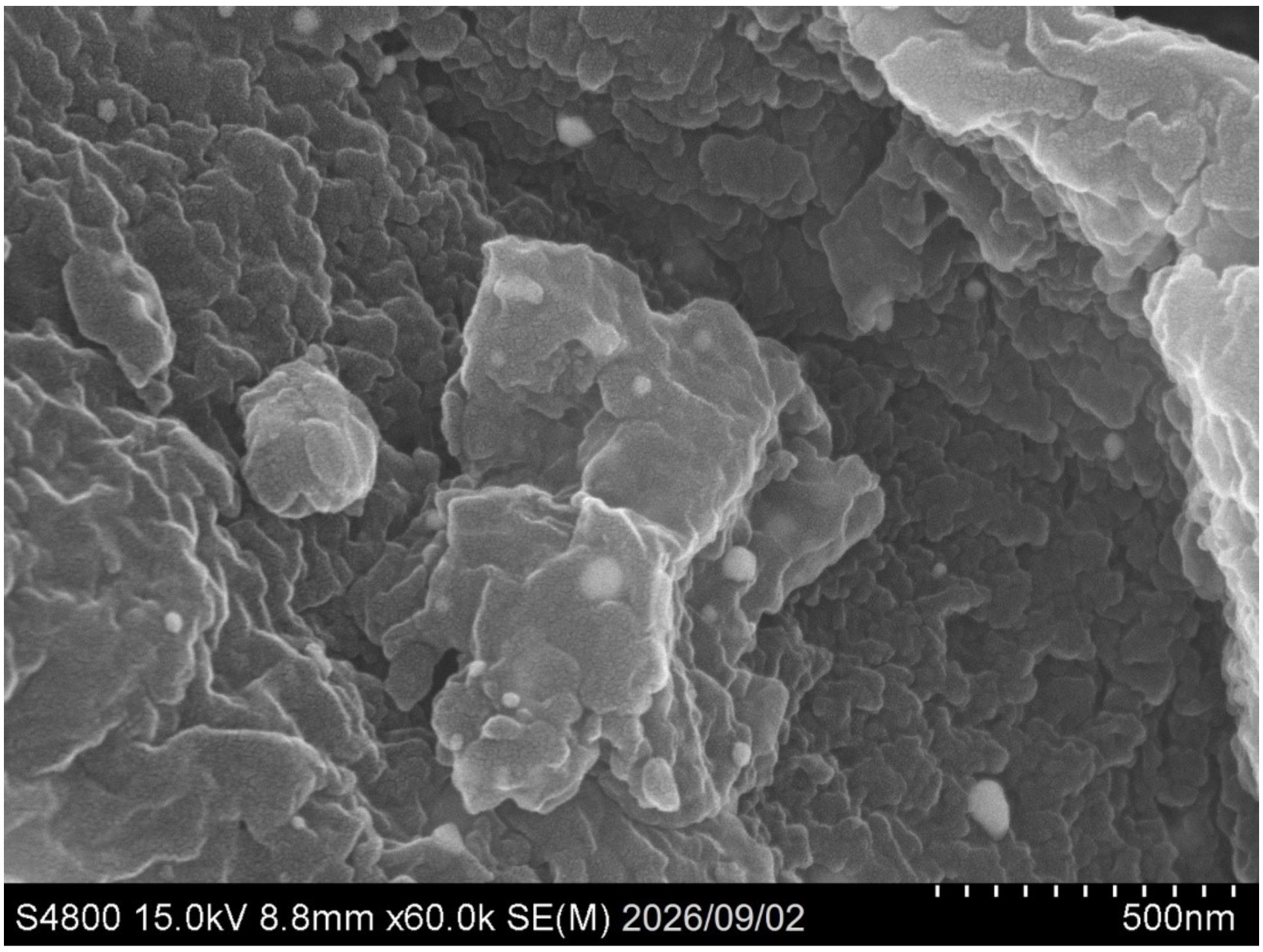

Figure S10: SEM Image of AtPdNP After 10th cycle of reuse

- **SEM Image of Catalyst AtPdNP After 10 Cycles of Reusability**

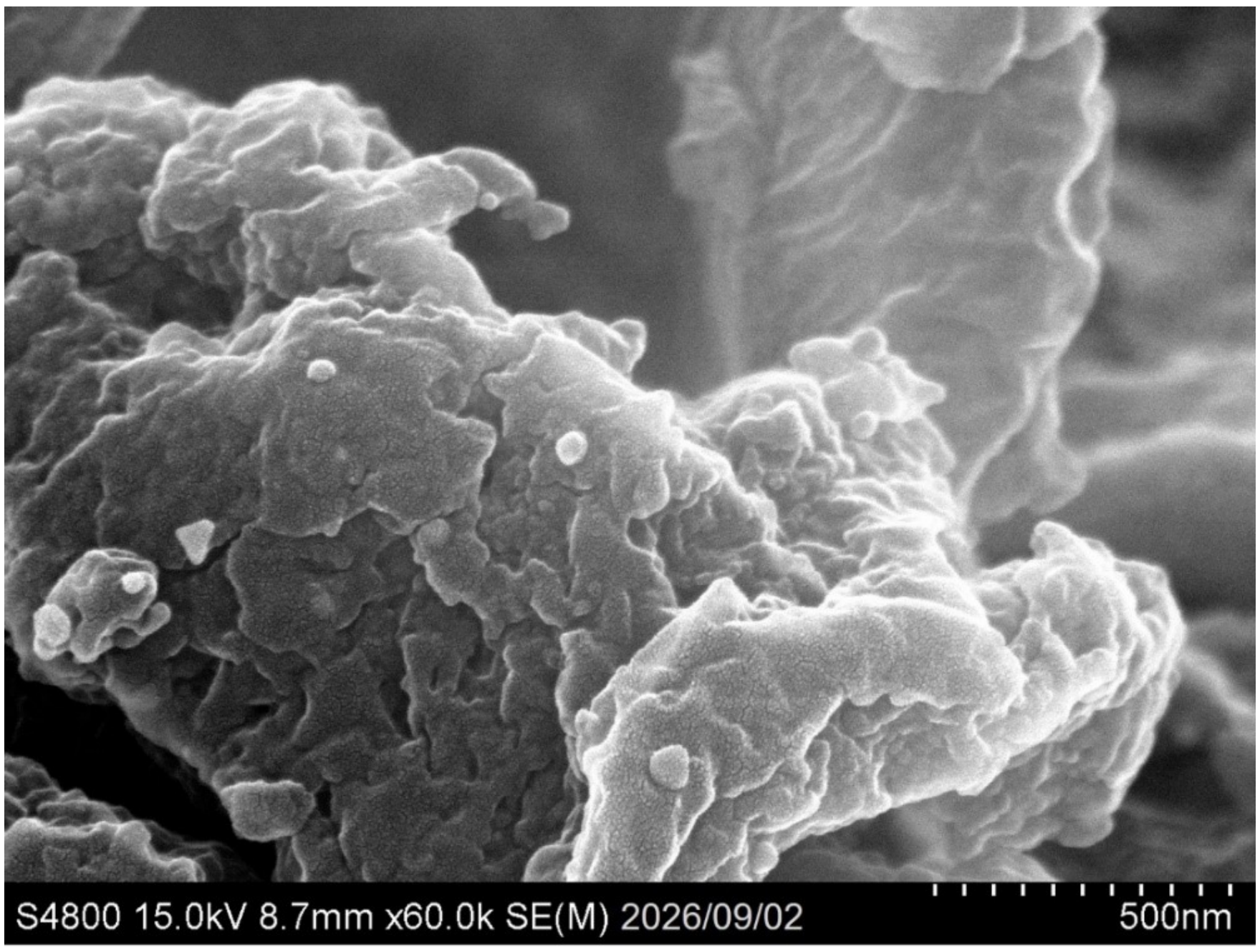

Figure S11: SEM Image of AtPdNP After 10 Cycles of Reusability

9 NMR For Substrate Scope

Structure	NMR Data	Ref
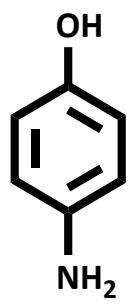	1H NMR (DMSO-d6; 500 MHz; δ in ppm): 8.33 (s, 1H), 6.47 (dd, 2H, J = 3, 9 Hz), 6.41 (dd, 2H, J = 3, 8.5 Hz), 4.40 (br s, 2H, D_2O exchanged); 13C NMR (DMSO-d6; 125 MHz; δ in ppm): 148.69, 141.08, 115.99, 115.71	[2]
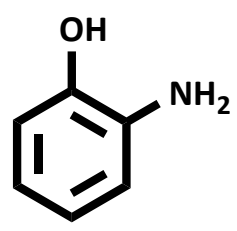	1H NMR (DMSO-d6; 500 MHz; δ in ppm): 7.71 (dd, 1H, J = 1.5, 8.0 Hz), 7.50 (dd, 1H, J = 1.5, 8.0 Hz), 7.46 (dt, 1H, J = 1.5, 8.0 Hz), 7.39 (dt, 1H, J = 1.5, 6.5 Hz), 6.82 (br s, 2H, D_2O exchanged), 6.36 (s, 1H, D_2O exchanged); 13C NMR (DMSO-d6; 125 MHz; δ in ppm): 155.2, 137.6, 133.6, 125.0, 120.2, 1198	[3]
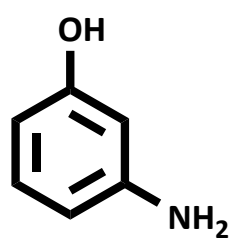	1H NMR (DMSO-d6; 500 MHz; δ in ppm): 8.77 (s, 1H), 6.71 (t, 1H, J = 8 Hz), 6.08 (s, 1H), 5.95 (d, 1H, J = 7.5 Hz), 5.87 (d, 1H, J = 7 Hz), 4.82 (br s, 2H, D_2O exchanged); 13C NMR (DMSO-d6; 125 MHz; δ in ppm): 163.27, 155.04, 134.61, 110.60, 108.48, 106.15	[4]
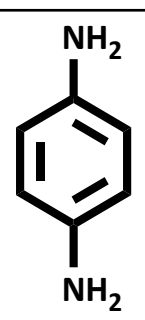	1H NMR ($CDCl_3$; 500 MHz; δ in ppm): 6.59 (s, 4H), 3.35 (br s, 4H, D_2O exchanged); 13C NMR ($CDCl_3$; 125 MHz; δ in ppm): 138.60, 116.73	[5]
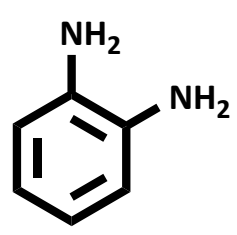	1H NMR ($CDCl_3$; 500 MHz; δ in ppm): 6.74 (m, 4H, J = 1 Hz), 3.40 (br s, 4H, D_2O exchanged); 13C NMR ($CDCl_3$; 125 MHz; δ in ppm): 134.74, 120.29, 116.76	[6]
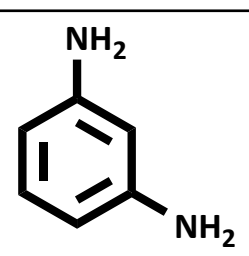	1H NMR ($CDCl_3$; 500 MHz; δ in ppm): 6.66 (s, 1H), 5.78 (m, 3H), 4.64 (br s, 4H); 13C NMR ($CDCl_3$; 125 MHz; δ in ppm): 149.81, 128.10, 104.15, 100.96	[7]

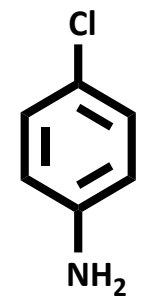	1H NMR (DMSO-d6; 500 MHz; δ in ppm): 7.01 (dd, 2H, J = 2.5, 8.5 Hz), 6.56 (dd, 2H, J = 2.5, 8.5 Hz), 5.21 (br s, 2H, D_2O exchanged); 13C NMR (DMSO-d6; 125 MHz; δ in ppm): 148.09, 128.95, 119.22, 115.66	[8]
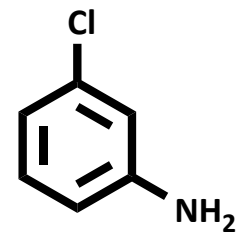	1 H NMR: δ 3.66 (s, br, 2H), 6.46-6.48 (m, 1H), 6.60 (t, J = 2.0 Hz, 1H), 6.69 (dd, J = 8.0, 2.0 Hz, 1H), 7.01(t, J = 8.0 Hz, 1H). 13C NMR: δ 113.3, 114.9, 118.4, 130.4, 134.8, 147.8.	[9]
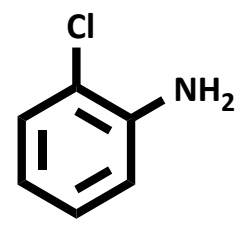	1H NMR (DMSO-d6; 500 MHz; δ in ppm): 7.17 (d, 1H, J = 10 Hz), 7.01 (t, 1H, J = 10 Hz), 6.78 (d, 1H, J = 8 Hz), 6.53 (t, 1H, J = 10 Hz), 5.30 (br s, 2H, D_2O exchanged); 13C NMR (DMSO-d6; 125 MHz; δ in ppm): 145.12, 129.41, 128.10, 117.51, 117.24, 115.00	[10]
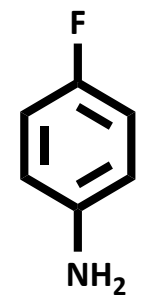	1H NMR (300 MHz, $CD3COCD_3$): d 5.30 (bs, 2H), 7.50 (s, 2H), 7.69 (s, 2H), 13C NMR (75 MHz, $CD3COCD_3$): d 115.8, 116.2, 145.6, 154.8, 157.9	[11]
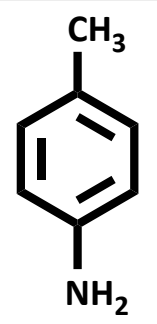	1H NMR (DMSO-d6, 500 MHz; δ in ppm): 6.81 (d, 2H, J = 8 Hz), 6.46 (d, 2H, J = 6.5 Hz), 4.72 (br s, 2H, D_2O exchanged), 2.12 (s, 3H); 13C NMR (DMSO-d6; 125 MHz; δ in ppm): 146.52, 129.68, 124.38, 114.50, 20.58	[12]
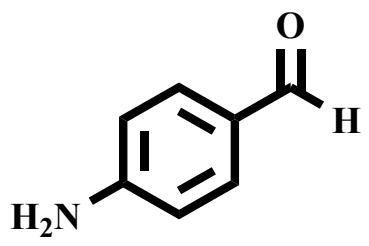	1H NMR ($CDCl_3$; 500 MHz; δ in ppm): 7.16 (d, 2H, J = 8.5 Hz), 6.68 (d, 2H, J = 8 Hz), 4.54 (s, 2H); 13C NMR ($CDCl_3$; 125 MHz; δ in ppm): 146.01, 131.08, 128.79, 115.19, 65.24	[2]
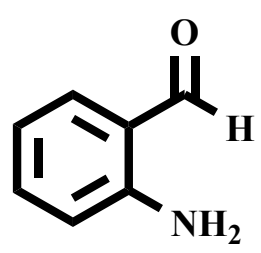	1H NMR ($CDCl_3$; 500 MHz; δ in ppm): 7.15 (dt, 1H, J = 1.5, 7.5 Hz), 7.07 (d, 1H, J = 7.5 Hz), 6.74 (dt, 1H, J = 1, 7.5 Hz), 6.71 (d, 1H, J = 7.5 Hz), 4.63 (s, 2H); 13C NMR ($CDCl_3$; 125 MHz; δ in ppm): 145.96, 129.34, 129.21, 124.94, 118.24, 116.10, 64.21	[13]
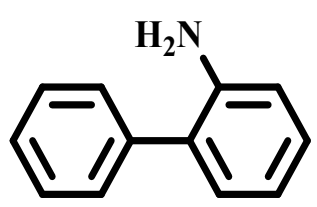	1 H NMR: δ 3.71 (s, br, 2H), 6.72-6.83 (m, 2H), 7.11-7.19 (m, 2H), 7.30-7.44 (m, 5H). 13C NMR: δ 115.6, 118.6, 127.2, 127.6, 128.5, 128.9, 129.1, 130.5, 139.6, 143.5	[14]
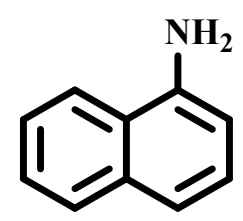	1H NMR (300 MHz, CDCl3): d 4.12 (bs, 2H), 6.81 (d, J = 6.084 Hz, 1H), 7.33-7.51 (m, 3H), 7.75-7.84 (m, 2H), 13C NMR (75 MHz, CDCl3): d 110.1, 119.4, 121.2, 124.1, 125.3, 126.3, 126.8, 129.0, 134.8, 142.4	[11]
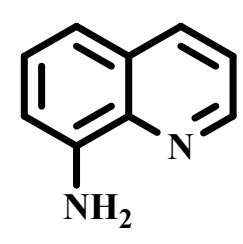	^{1}H-NMR (500 MHz, $CDCl_3$) δ = 8.77 - 8.75 (dd, *J* = 5 Hz, 1H), 8.08 - 8.06 (dd, *J* = 5 Hz, 1H), 7.38 - 7.32 (m, 2H), 7.16 - 7.14 (dd, *J* = 10 Hz, 1H), 6.94 - 6.92 (dd, *J* = 10 Hz, 1H), 4.88 (s, 2H) ppm; ^{13}C-NMR (125 MHz, $CDCl_3$) δ = 147.1, 143.7, 137.9, 136.0, 128.7, 127.3, 121.1, 115.86, 110.0	[15]

10 References

1. Rathod, H.B., et al., *Palladium Bio-Nanocomposite as an Efficient Heterogeneous Catalyst for Nitro Reduction: Fungus Mediated Green and Sustainable Process.* Nanoscale Advances, 2026.

2. Ge, Q., et al., *Selective reduction of aromatic nitro compounds over recyclable hollow fiber membrane-supported A u nanoparticles.* Journal of Applied Polymer Science, 2015. **132**(2).
3. Karthik, M. and P. Suresh, *Greener Synthesis of Reduced Graphene Oxide-Nickel Nanocomposite: Rapid and Sustainable Catalyst for the Reduction of Nitroaromatics.* ChemistrySelect, 2017. **2**(23): p. 6916-6928.
4. Layek, K., et al., *Gold nanoparticles stabilized on nanocrystalline magnesium oxide as an active catalyst for reduction of nitroarenes in aqueous medium at room temperature.* Green chemistry, 2012. **14**(11): p. 3164-3174.
5. Jia, W.-G., et al., *Half-sandwich ruthenium complexes with Schiff-base ligands: Syntheses, characterization, and catalytic activities for the reduction of nitroarenes.* Organometallics, 2016. **35**(4): p. 503-512.
6. Nabid, M.R., et al., *Yolk/shell AuNPs@ polyethyleneimine-derived carbon nanoparticles as nanoreactor for catalytic nitroarenes reduction.* Catalysis Letters, 2016. **146**(1): p. 229-237.
7. Rao, H., et al., *Easy Copper-catalyzed synthesis of primary aromatic amines by couplings aromatic boronic acids with aqueous ammonia at room temperature.* Angewandte Chemie, 2009. **121**(6): p. 1134-1136.
8. Guo, Y., et al., *Palladium-modified functionalized cyclodextrin as an efficient and recyclable catalyst for reduction of nitroarenes.* RSC advances, 2016. **6**(10): p. 7950-7954.
9. McLaughlin, M.A. and D.M. Barnes, *A practical and selective reduction of nitroarenes using elemental sulfur and mild base.* Tetrahedron letters, 2006. **47**(51): p. 9095-9097.
10. Motoyama, Y., K. Kamo, and H. Nagashima, *Catalysis in polysiloxane gels: platinum-catalyzed hydrosilylation of polymethylhydrosiloxane leading to reusable catalysts for reduction of nitroarenes.* Organic letters, 2009. **11**(6): p. 1345-1348.
11. Sharma, U., et al., *Highly chemo-and regioselective reduction of aromatic Nitro compounds catalyzed by recyclable Copper (II) as well as Cobalt (II) phthalocyanines.* Advanced Synthesis & Catalysis, 2010. **352**(11-12): p. 1834-1840.
12. Dabiri, M., N.F. Lehi, and S.K. Movahed, *Fe3O4@ RGO@ Au@ C Composite with Magnetic Core and Au Enwrapped in Double-Shelled Carbon: An Excellent Catalyst in*

the Reduction of Nitroarenes and Suzuki–Miyaura Cross-Coupling. Catalysis Letters, 2016. **146**(9): p. 1674-1686.

13. Vasilikogiannaki, E., et al., *Facile reduction of nitroarenes into anilines and nitroalkanes into hydroxylamines via the rapid activation of ammonia· borane complex by supported gold nanoparticles.* Advanced Synthesis & Catalysis, 2013. **355**(5): p. 907-911.
14. Xu, H. and C. Wolf, *Efficient copper-catalyzed coupling of aryl chlorides, bromides and iodides with aqueous ammonia.* Chemical communications, 2009. **45**(21): p. 3035-3037.
15. Singh, H., et al., *Magnetically separable and reusable Fe3O4 nanoparticles for reduction of nitroarenes in water.* ChemistrySelect, 2025. **10**(14): p. e202501004.
